# Molecular Basis for Polyketide Ketoreductase–Substrate Interactions

**DOI:** 10.3390/ijms21207562

**Published:** 2020-10-13

**Authors:** Shiji Zhao, Fanglue Ni, Tianyin Qiu, Jacob T. Wolff, Shiou-Chuan Tsai, Ray Luo

**Affiliations:** 1 Departments of Molecular Biology and Biochemistry, Chemical and Biomolecular Engineering, Materials Science and Engineering, and Biomedical Engineering, University of California, Irvine, CA 92697, USA; shijiz@uci.edu (S.Z.); fangluen@uci.edu (F.N.); tianyiq1@uci.edu (T.Q.); 2 Departments of Molecular Biology and Biochemistry, Chemistry, and Pharmaceutical Sciences, University of California, Irvine, CA 92697, USA; jwolff1@uci.edu

**Keywords:** natural products, ketoreductase, polyketides, computational chemistry, molecular dynamics

## Abstract

Polyketides are a large class of structurally and functionally diverse natural products with important bioactivities. Many polyketides are synthesized by reducing type II polyketide synthases (PKSs), containing transiently interacting standalone enzymes. During synthesis, ketoreductase (KR) catalyzes regiospecific carbonyl to hydroxyl reduction, determining the product outcome, yet little is known about what drives specific KR–substrate interactions. In this study, computational approaches were used to explore KR–substrate interactions based on previously solved apo and mimic cocrystal structures. We found five key factors guiding KR–substrate binding. First, two major substrate binding motifs were identified. Second, substrate length is the key determinant of substrate binding position. Third, two key residues in chain length specificity were confirmed. Fourth, phosphorylation of substrates is critical for binding. Finally, packing/hydrophobic effects primarily determine the binding stability. The molecular bases revealed here will help further engineering of type II PKSs and directed biosynthesis of new polyketides.

## 1. Introduction

Polyketides form a large family of natural products with a diverse array of chemical structures and bioactivities [1]. Many polyketides have important pharmaceutical properties and can be used as anticancer, antibiotic, and antihypercholesterol drugs [2,3,4]. In nature, polyketides are biosynthesized by multi-enzyme complexes called polyketide synthases (PKSs) in plants, fungi and bacteria. Because of their medical importance, there has been a vigorous effort to engineer PKSs to produce new polyketides with therapeutic potential [1]. PKSs are genetically, structurally, and enzymatically homologous to fatty acid synthases (FASs) [5], and are categorized into three types based on their architectures: type I, type II, and type III [6]. This study focuses on reducing type II PKSs found mostly in bacteria, whose products are aromatic polyketides such as actinorhodin [2]. Reducing type II polyketide biosynthesis proceeds through four common steps: (1) chain elongation, catalyzed by ketosynthase/chain length factor (KS/CLF); (2) regiospecific reduction, catalyzed by ketoreductase (KR); (3) aromatization/cyclization, catalyzed by aromatase/cyclase (ARO/CYC); and (4) system-specific chemical modification, carried out by a variety of other enzymes [7]. During the entire process, the growing polyketide chain is covalently attached to the acyl carrier protein (ACP) at the conserved active site serine, using the phosphopantetheine linker [8]. In total, polyketide production can involve more than 20 enzyme-catalyzed reactions to produce one major product. It is the controlled selection by PKS of starter units, chain length, reduction and cyclization patterns that result in the huge diversity of polyketides observed in nature.

Many polyketide engineering attempts have tried to take advantage of the discrete nature of each step to mix and match proteins from different systems to produce novel products. Understanding the molecular factors controlling selection is needed to successfully engineer PKSs that synthesize “unnatural” natural products that can be developed into new therapeutics. Despite past research into type II PKS synthesis, how KS/CLF, ACP, and KR choreograph their respective reactions while maintaining precise chain length, regio-, and stereo-specificity remains a mystery. Such a lack of knowledge has greatly hampered type II polyketide engineering efforts [1]. Therefore, there is a need to understand the molecular basis for the chain length and regiospecificity observed in type II PKSs. This work focuses on elucidating the binding mechanism of the poly-β-ketone intermediate with the KR, which catalyzes the first carbonyl to hydroxyl reduction of a single carbon group to a hydroxyl group [9]. In addition, KR is also hypothesized to be able to catalyze first ring cyclization. However, it is highly selective in reducing polyketide with certain chain lengths [10,11]. Therefore, the study of the polyketide intermediates selection mechanism by KR is essential to understand how PKS controls its product outcome.

**Figure 1:**
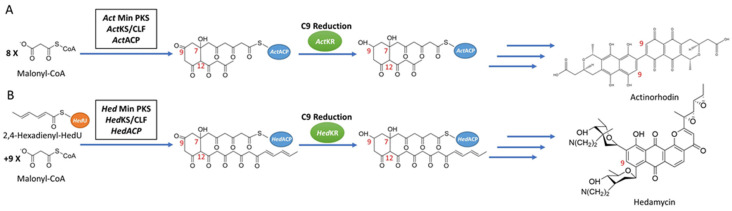
Synthesis pathways of reducing type II PKSs, using hedamycin and actinorhodin as examples. (**A**) The synthesis pathway of actinorhodin, in which C9 reduction of the 16-carbon poly-β-ketone intermediate is catalyzed by *Act*KR. (**B**) The synthesis pathway of hedamycin, in which C9 reduction of the 24-carbon poly-β-ketone intermediate is catalyzed by *Hed*KR. Abbreviations: ACP, acyl carrier protein; CLF, chain length factor; CoA, coenzyme A; KR, ketoreductase; KS, ketosynthase; PKS, polyketide synthase.

In this study, chain length specificity distinct actinorhodin KR (*Act*KR) and hedamycin KR (*Hed*KR) were used as model KRs (Figure 1). Actinorhodin is a pigmented antibiotic produced by a type II PKS from *Streptomyces coelicolor* [7,9], and hedamycin is a pluramycin-type antitumor antibiotic produced by *Streptomyces griseoruber* [12,13]. The actinorhodin PKS is the model system of type II PKS, and the first type II KR structure reported was *Act*KR co-crystallized with the cofactor NADPH [9]. *Act*KR specifically reduces the C9 carbonyl group of a 16-carbon poly-β-ketone intermediate [14]. In contrast, *Hed*KR is able to reduce tetra-, octa-, undeca-, and dodeca-ketides [15,16]. *Act*KR and *Hed*KR has high sequence homology (61% sequence identity), and both KRs specifically reduce the C9 carbonyl group [17]. It remains a mystery how *Hed*KR may have higher promiscuity in terms of chain length control than that of *Act*KR. Based on sequence alignment (Figure S1) in and around the KR active site, H153 and H201 are conserved among many type II KRs but not *Hed*KR, which has tyrosine and glycine at these two positions. This difference led us to hypothesize that these residues could be responsible for controlling what length of poly-β-ketone intermediates could successfully enter the active site. To test our hypothesis, we created a H153Y/H201G double mutant *Act*KR, with the expectation that DM-*Act*KR will have similar promiscuity as *Hed*KR. (Manuscript in preparation).

**Figure 2:**
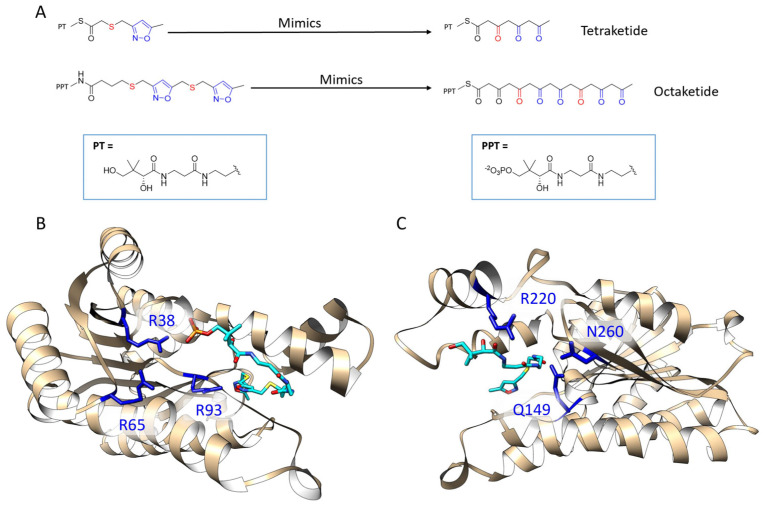
Previously solved co-crystals of double mutant *Act*KR (DM-*Act*KR) bound with isoxazole-based linear poly-β-ketone mimics revealed two potential substrate-binding residue patches (monomers shown). (**A**) Pantetheinylated (PT) tetraketide (8 carbons) and phosphopantetheinylated (PPT) octaketide (16 carbons) mimics synthesized to probe PKS active sites. Sulfur and isoxazole substitutions to replace the native carbonyls are displayed in red and blue, respectively. (**B**) DM-*Act*KR-octaketide-PPT co-crystal structure indicated the mimic’s phosphate bound to a “front-patch”: R38, R65, R93. (**C**) DM-*Act*KR-tetraketide-PT co-crystal structure showed interactions between PT and a “back-patch”: Q149, R220, N260. Mimics are displayed in cyan; patch residues are displayed in blue.

A persisting problem of type II PKS research is that the polyketide intermediates of type II PKS are highly reactive, which are apt to have spontaneous aldol cyclization, resulting in great difficulty to isolate the intermediates and use them experimentally for X-ray crystallography studies or enzymological analysis [18]. To fully understand the binding mechanism of the KR with its polyketide intermediates, a series of stable isoxazole-based polyketide mimics were synthesized [19]. These mimics substituted some of the polyketide carbonyl groups with sulfur and isoxazole to achieve stability (Figure 2A). After extensive crystallization effort of both wild type and double mutant *Act*KRs with the mimic probes, we were able to crystallize and solve the co-crystal structures of the double mutant (DM-*Act*KR) bound with tetraketide-pantetheine and octaketide-phosphopantetheine mimics, which were used as templates for computational studies in this work.

The positions of the polyketide substrates binding raise an interesting question. In the structure of DM-*Act*KR bound with the octaketide-phosphopantetheine mimic, the phosphate group binds closely to previously proposed positively-charged arginine patch (defined as the front-patch), which is formed by a cluster of arginine residues (R38, R65, R93) that interact with the phosphate group of the phosphopantetheine moiety of the incoming polyketide intermediate (Figure 2B) [9]. However, in the structure bound with the tetraketide-pantetheine mimic, the pantetheine moiety is close to another cluster of positive and amidic residues (Q149, R220, N260), which was defined as the back-patch (Figure 2C). It would be of great interest to analyze if polyketide intermediates with different chain lengths and with or without phosphorylation would bind in a different position, and if different KR conformation causes any change in the binding motif.

In this work, we applied molecular dynamics (MD) simulations to investigate the polyketide binding mechanism from two perspectives. First, the effect of polyketide length or substrate phosphorylation on the binding orientation of polyketide intermediates (front-patch or back-patch). Second, the effect of a double mutation on *Act*KR on polyketide binding. To evaluate if isoxazole-based mimics are comparable with the actual polyketide intermediates, MD simulations were conducted using both the actual polyketide intermediates and the polyketide mimics. The results from MD simulations help us understand how KR recognizes polyketide intermediates with different chain lengths, which will help further engineering of type II PKS and the directed biosynthesis of new polyketides.

## 2. Results

### 2.1. Fragment Docking Identified Front- and Back-Patches as Two Major Binding Motifs

**Figure 3:**
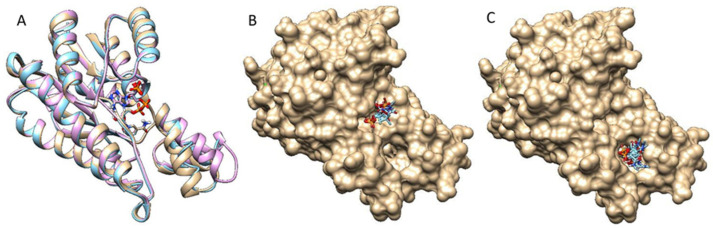
DM-*Act*KR monomer conformation comparison and two major binding motif clusters generated from docking analysis on octaketide-bound DM-*Act*KR monomer (closed conformation). (**A**) Visualization of aligned monomers among the structures originally containing: octaketide-bound (gold), tetraketide-bound (cyan), and no ligand (pink). The NADPH present in all three structures is displayed; ligands deleted for clarity. (**B**) The front-patch binding motif. (**C**) The back-patch binding motif. The protein and NADPH surface are displayed in gold.

Three DM-*Act*KR crystal structures were used as docking templates for molecular docking analysis: a previously solved DM-*Act*KR co-crystal structure bound with the octaketide-phosphopantetheine mimic, a DM-*Act*KR co-crystal structure bound with the tetraketide-pantetheine mimic, and an apo structure of DM-*Act*KR mutated *in silico* from WT-*Act*KR (Protein Data Bank—PDB ID: 1X7H). In particular, the monomer subunit that contains the substrate mimic in each tetrameric cocrystal structure was used. Three major conformations are present in the three DM-*Act*KR structures used as templates: closed, half-closed, and open conformations, corresponding to octaketide mimic-bound, tetraketide mimic-bound, and apo structures, respectively (Figure 3A). This trend in conformational variation between different ligands could be explained by the fact that a larger ligand can form more protein–ligand interactions and create a stronger ligand–enzyme interaction, leading to a more closed conformation.

In order to enhance the exhaustiveness and specificity of the docking analysis by limiting the amount of degrees of freedom on conformation space, a fragment that contains the entire phosphate group and a part of pantetheine were used as the docking ligand (Figure S2). All three conformations were docked with the fragment for 10,000 independent rounds, and the first 200 binding modes with the highest scores were analyzed. The docking results reveal two major binding motifs in all three conformations, which are consistent with the previously identified front-patch and back-patch (Figure 3B,C). There are a few other sites detected from the docking result, but they are either buried under the tetrameric interface, or the frequency is too low to be considered significant. In the open conformation, all binding poses are located at the back-patch, indicating that the open conformation provides a highly exposed binding pocket that the probe can bind to instead of the front-patch. In the analysis of the half-closed conformation docking simulation, 3.3% of the poses were at the front-patch and 96.0% were at the back-patch. This trend is repeated in the closed conformation docking analysis, with 1.0% front-patch poses and 98.0% back-patch poses. In total, 98.0% of high-scoring binding poses appear at the back-patch regardless of conformation. This reveals the trend that binding pockets in the closed and half-closed forms tend to accept more ligands in front-patch binding poses (Figure 3B), which can be explained by a narrower back-patch binding site in combination with a wider front-patch binding site in the closed conformation binding pocket.

### 2.2. Pantetheine or Phosphopantetheine Moiety Is Necessary for Ligand Binding

Twenty-four DM-*Act*KR-ligand complexes were prepared for MD simulations through structure alignment using the two DM-*Act*KR co-crystal structures solved previously as templates (Table 1), among which DM-*Act*KR-(m-oct-pp) (ligand binds to front-patch) and DM-*Act*KR-(m-tet-p) (ligand binds to back-patch) are experimental structures. A framewise stability score (SS) was developed as a measure to evaluate the binding stability of each KR-ligand pair, with SS close to zero indicating weak binding, and SS close to one indicating strong binding (See Supporting Information A.4 for detail). Each system was simulated in triplicate using identical minimized structures. 200 ns MD simulation were performed on each minimized structure, with root-mean-square deviation (RMSD) and SS plots showing that all trajectories had reached equilibrium by 100 ns (Figure S3). Surprisingly, all ligands without a pantetheine or phosphopantetheine moiety (m-tet, tet, m-oct, oct) exited the DM-*Act*KR binding pocket within 200 ns, regardless of initial binding position. These results strongly indicate that pantetheine or phosphopantetheine are essential for KR-ligand binding for any polyketide or polyketide mimic and might explain why none of our previous attempts to co-crystalize KRs with mimics lacking these moieties have ligand electron density. Thus, for further simulations, only ligands with pantetheine or phosphopantetheine moiety were prepared.

### 2.3. Polyketide Length Determines Ligand Binding Position

As discussed above, all ligands without pantetheine or phosphopantetheine do not remain bound in the DM-*Act*KR binding pocket. Therefore, there are only 16 DM-*Act*KR-ligand complexes left to be considered from Table 1. Because two potential binding sites (front-patch and back-patch) have been revealed by previous experimental structures, we investigated what key factor(s) determines the ligand binding site, i.e., given a specific polyketide ligand, which binding site the ligand would go to.

**Figure 4:**
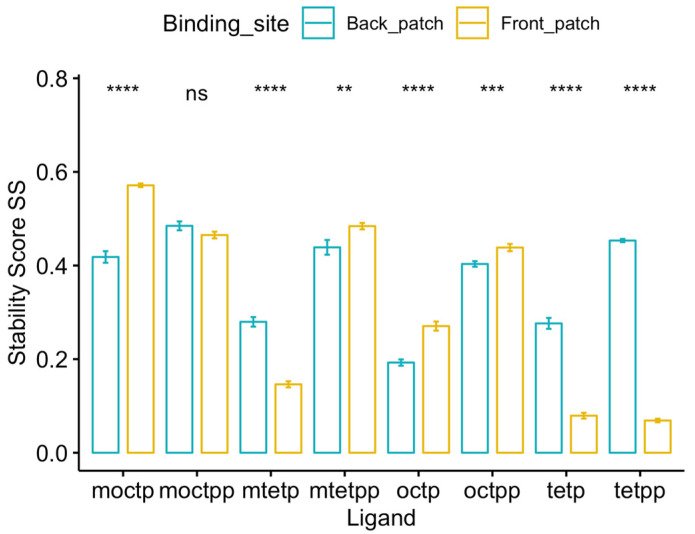
Stability score SS analysis of 8 ligands bound to front-patch and back-patch binding positions of DM-*Act*KR. Among all 4 octaketide ligands, 3 of them (m-oct-p, oct-pp, oct-p) showed a significantly higher SS at front-patch. While among all 4 tetraketide ligands, 3 of them (m-tet-p, tet-pp, tet-p) show significantly higher SS at back-patch. Significance levels: ns, *p* > 0.05; *, *p* <= 0.05; **, *p* <= 0.01; ***, *p* <= 0.001; ****, *p* <= 0.0001.

The two previously solved co-crystal structures have shown that the two mimics bind to the binding pocket of DM-*Act*KR at different sites. The phosphopantetheine moiety of m-oct-pp binds to the front-patch, while the pantetheine moiety of m-tet-p binds to the back-patch. There are two major differences between the m-oct-pp and m-tet-p mimics: polyketide length (16 and eight carbons) and pantetheine phosphorylation (phosphorylated and not phosphorylated). It is reasonable to assume that one of the two factors determine the ligand binding position. The stability score SS of the last 100 ns of each trajectory were extracted and compared pairwise, grouped by ligand identity. Three of four octaketide ligands (m-oct-p, oct-pp, oct-p) showed higher average SS towards front-patch, while three of four tetraketide ligands (m-tet-p, tet-pp, tet-p) showed higher average SS towards back-patch (Figure 4). This indicates that polyketide length is a consistent and significant factor determining ligand binding site. Conversely, pantetheine phosphorylation is not significantly correlated with a specific ligand binding site. Surface visualization of the DM-*Act*KR binding pocket shows that the front-patch (R38, R65, R93) and the back-patch (Q149, R220, N260) form two opposite entrances of a long channel, in which the active site catalytic residues (N114, S144, Y157, K161) are located at the center (Figure S4). Shorter polyketide substrates, such as a tetraketide, may enter the active site more easily through the back-patch compared to longer substrates.

### 2.4. ActKR H153Y/H201G Double Mutation Increases Ligand Binding Affinity

Histidines 153 and 201 near the *Act*KR active site were identified as potentially enforcing a minimum chain length based on conservation with other KRs and substitutions at those positions in the apparently more promiscuous *Hed*KR (Figure S1) [15,20]. Thus, a H153Y/H201G DM-*Act*KR was generated to test the hypothesis that DM-*Act*KR will show higher binding affinity towards polyketides with lengths that differ from *Act*KR’s canonical 16 carbon substrate. Eight new KR-ligand complexes were prepared through structural alignment, including four WT-*Act*KR-ligand complexes as negative controls and four WT-*Hed*KR-ligand complexes as positive controls (Table 2). The structure of WT-*Act*KR was prepared by mutating Y153 and G201 of the DM-*Act*KR cocrystal structure to histidine, and the WT-*Hed*KR structure was obtained from the Protein Data Bank (PDB ID: 3SJU). All octaketide ligands were aligned to the front-patch, and all tetraketide ligands to the back-patch, in line with the front/back-patch docking results.

**Figure 5:**
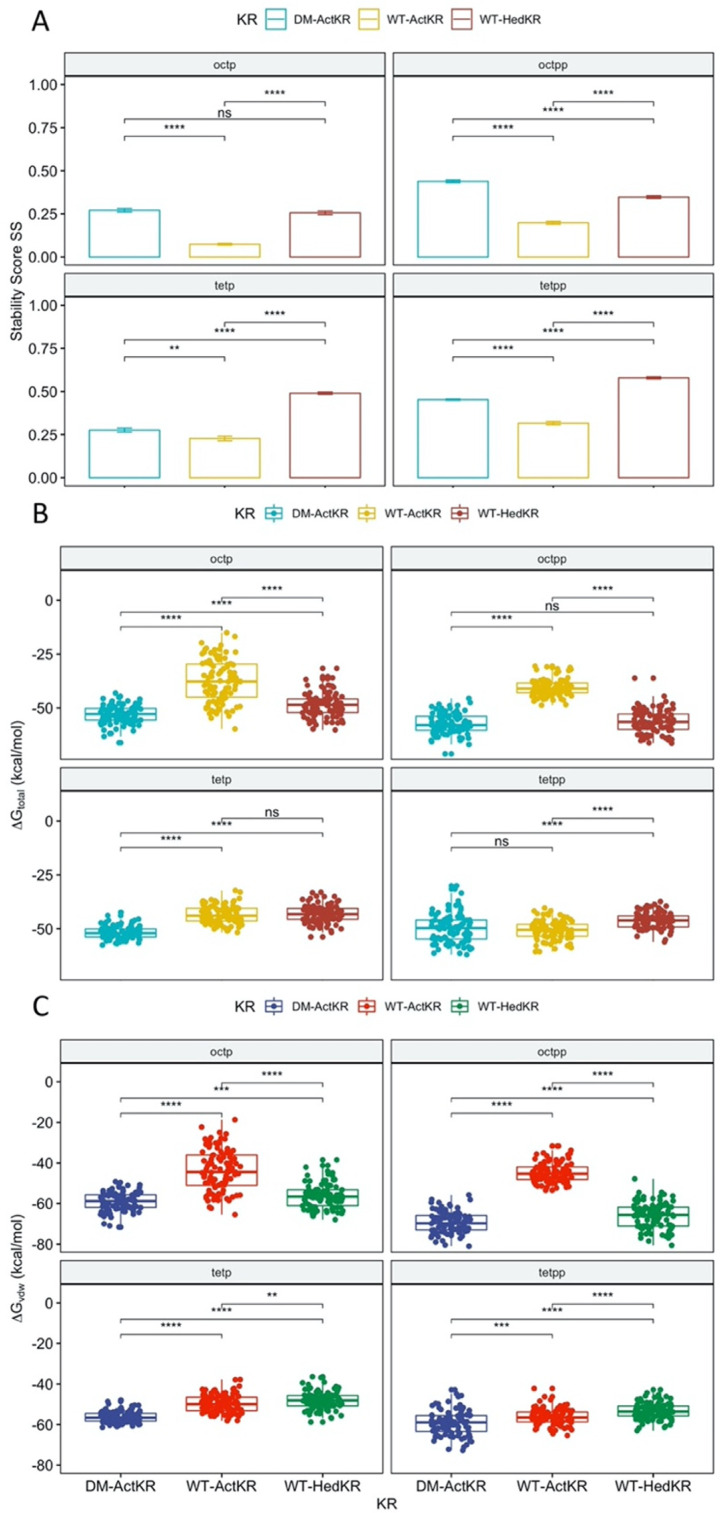
Stability score SS analysis and MMPBSA comparison of DM-*Act*KR, WT-*Act*KR and WT-*Hed*KR bound with tetraketides and octaketides. (**A**) For each ligand, the average stability score SS of DM-*Act*KR is significantly increased compared with WT-*Act*KR and is closer to that of WT-*Hed*KR. (**B**) The total binding free energy $\Delta G_{total}$ results. The total binding free energy of DM-*Act*KR is shifted towards that of WT-*Hed*KR for octaketides, but not tetraketides. (**C**) Non-electrostatic binding free energy $\Delta G_{vdw}$ shows similar pattern as (**B**). Significance levels: ns, *p* > 0.05; *, *p* <= 0.05; **, *p* <= 0.01; ***, *p* <= 0.001; ****, *p* <= 0.0001.

RMSD plots as well as SS plots show that all trajectories had reached equilibrium after 100 ns (Figure S3). Therefore, the SS of the last 100 ns of each trajectory were extracted for *t*-test analysis, grouped by KR type (Figure 5A). For each ligand, the DM-*Act*KR-ligand complexes showed significantly higher average SS than the corresponding WT-*Act*KR-ligand complexes, and the average DM-*Act*KR-ligand complex SS’s are closer to the corresponding average WT-*Hed*KR-ligand complex SS’s than the average WT-*Act*KR-ligand complex SS’s.

Furthermore, MMPBSA analysis was performed to complement the stability score analysis [21,22,23,24,25]. Among the three trajectories simulated for each KR-ligand complex, the total binding free energy, $\Delta G_{total}$, non-electrostatic binding free energy, $\Delta G_{vdw}$, and electrostatic binding free energy, $\Delta G_{ele}$, from the last 100 ns of the trajectory with the highest average SS, were used for MMPBSA *t*-test analysis, grouped by KR type. Non-electrostatic binding free energy $\Delta G_{vdw}$ reflects packing/hydrophobic effects of the system and is the sum of the VDWAALS (van der Waals energy change upon binding) and ENPOLAR terms (nonpolar solvation free energy change upon binding). Electrostatic binding free energy $\Delta G_{ele}$ reflects the electrostatic effects within the system and is the sum of the EEL (electrostatic energy change upon binding) and EPB terms (electrostatic solvation free energy change upon binding). The total binding free energy $\Delta G_{total}$ results show that the *Act*KR double mutation significantly reduces the binding free energy for octaketide ligands (oct-pp, oct-p), performing more similarly to WT-*Hed*KR than WT-*Act*KR (Figure 5). However, two interesting results were observed for the tetraketide ligands (tet-pp, tet-p).

First, while the binding energy of both ligands is close for the DM-*Act*KR, the tet-pp ligand has decreased binding energy with WT-*Act*KR compared to the tet-p ligand and is similar to the DM-*Act*KR binding energy. This shows that the presence of a phosphate group on the tetraketide counteracts the positive effect on the binding affinity caused by the double mutation on WT-*Act*KR. A possible explanation is the “hanging-chain effect” on linear ligand binding that occurs when both ends of the ligand are tightly constrained by the binding pocket (Figure S5). This leaves the linear moiety without many interactions with nearby residues, leading to weaker binding affinity compared with those ligands with only one end constrained. The second interesting point is that the total binding free energy of WT-*Hed*KR is not significantly lower than that of WT-*Act*KR as expected, indicating that *Hed*KR is not necessarily more promiscuous than *Act*KR. In fact, whether WT-*Act*KR can reduce short polyketide intermediates is still a debatable question, because it was observed that the same products were generated from the minimal hedamycin PKS (*Hed*KS/CLF and *Hed*ACP) when combined with WT-*Act*KR or *Hed*KR [17].

The non-electrostatic binding free energy $\Delta G_{vdw}$ results show nearly identical patterns as the total binding free energy, indicating that packing/hydrophobic effects are the main contributing factors to KR-substrates binding (Figure 5C). On the other hand, the electrostatic binding free energy $\Delta G_{ele}$ results show random patterns compared with the total binding free energy results (Figure S6). Framewise Pearson correlation tests show that for all 12 KR-ligand pairs, the total binding free energy $\Delta G_{total}$ has higher correlation with non-electrostatic binding free energy $\Delta G_{vdw}$, rather than electrostatic binding free energy $\Delta G_{ele}$ (Figure S7).

### 2.5. Phosphate Group Contributes to Ligand Binding through van der Waals Interactions

**Figure 6:**
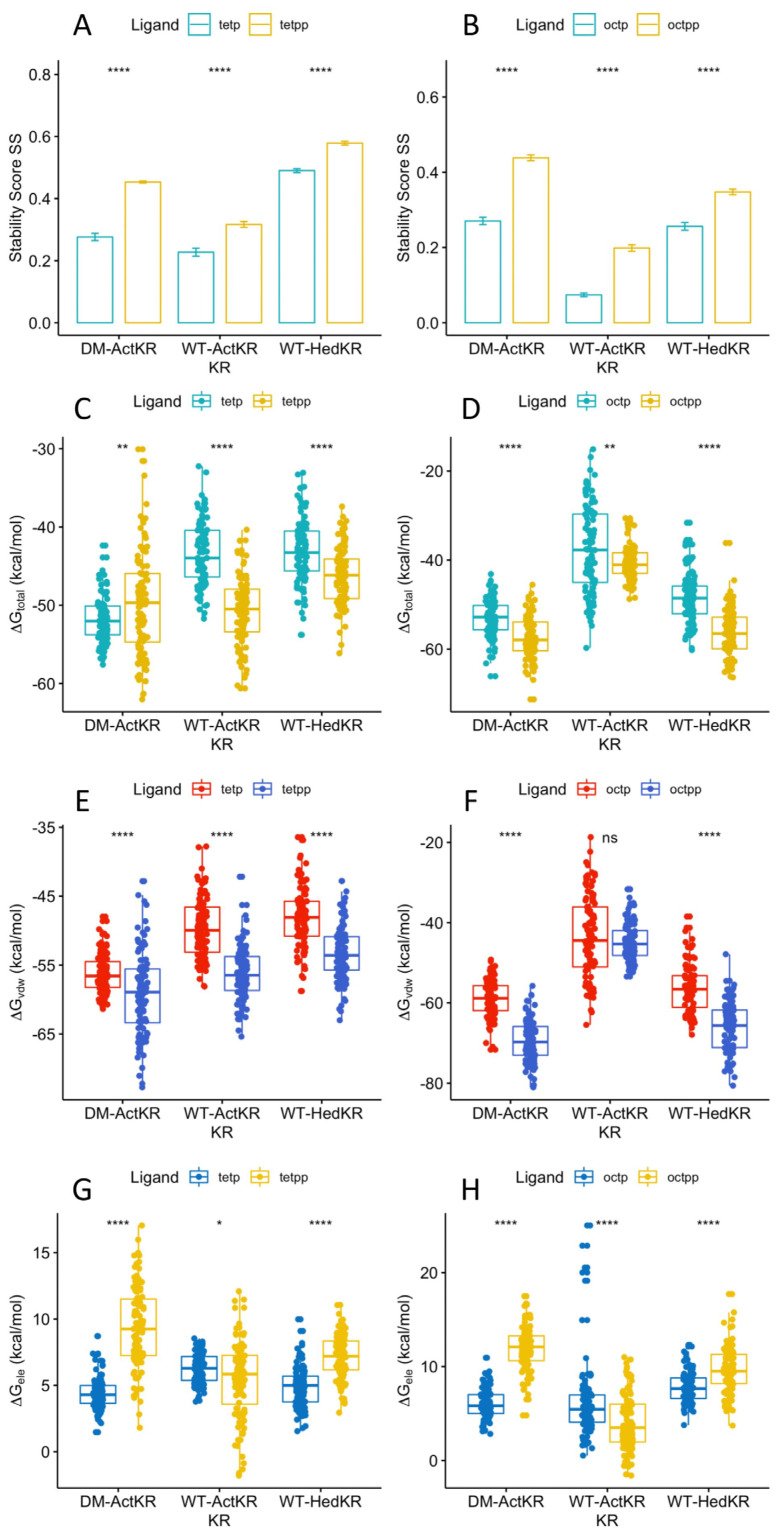
Stability score SS and MMPBSA comparison of DM-*Act*KR, WT-*Act*KR and WT-*Hed*KR bound with tetraketides and octaketides. All systems in (**A**,**B**) showed identical pattern that ligands with phospho-pantetheine moiety had significantly higher SS than those with pantetheine moiety, regardless of being octaketide or tetraketide. (**C**) Total binding free energy $\Delta G_{total}$ of tetraketides binding. (**D**) Total binding free energy $\Delta G_{total}$ of octaketides binding. (**E**) Non-electrostatic binding free energy $\Delta G_{vdw}$ of tetraketides binding. (**F**) Non-electrostatic binding free energy $\Delta G_{vdw}$ of octaketides binding. (**G**) Electrostatic binding free energy $\Delta G_{ele}$ of tetraketides binding. (**H**) Electrostatic binding free energy $\Delta G_{ele}$ of octaketides binding. Significance levels: ns, *p* > 0.05; *, *p* <= 0.05; **, *p* <= 0.01; ***, *p* <= 0.001; ****, *p* <= 0.0001.

Ligand positioning in the DM-*Act*KR-(m-oct-pp) and DM-*Act*KR-(m-tet-p) co-crystal structures provides grounds for the phosphate-front/back-patch interaction contributing significantly to the initial ACP-phosphopantetheine-polyketide and KR docking phase. A comparison of the stability score SS of ligands with pantetheine moiety or phosphopantetheine moiety grouped by polyketide type shows that the presence of the phosphate group significantly increases KR-ligand binding stability in each KR-ligand system (Figure 6A,B). In addition, the total binding free energy, $\Delta G_{total}$, from the trajectory with the highest average SS was analyzed for each KR-ligand system (Figure 6C,D). This analysis shows that for each ketoreductase system used, ligands with a phosphopantetheine moiety tend to have lower binding free energies than those with a pantetheine moiety, regardless of ligand length. The only exception is the DM-*Act*KR-tetraketide, where tet-pp binding free energy is higher than tet-p which might be due to the “hanging-chain effect” as mentioned earlier (Figure S5). The non-electrostatic binding free energy, $\Delta G_{vdw}$, results exhibit nearly identical patterns as the total binding free energy, $\Delta G_{total}$ (Figure 6E,F). It is worth noting that phosphorylated ligand electrostatic binding free energy $\Delta G_{ele}$ to WT-*Act*KR is consistently higher than unphosphorylated ligand, while ligand electrostatic binding free energy $\Delta G_{ele}$ to DM-*Act*KR and WT-*Hed*KR follows the opposite trend (Figure 6G,H). This indicates that the “chain length filter” mutation from histidine to tyrosine/glycine swapped *Act*KR’s electrostatic affinity for negatively charged phosphorylated ligands. Nonetheless, the high correlation coefficient between the total binding free energy $\Delta G_{total}$ and non-electrostatic binding free energy $\Delta G_{vdw}$ still suggests that the effect of van der Waals interactions of the phosphate group with the front/back-patch is greater than that of electrostatic interactions (Figure S7).

Therefore, the packing/hydrophobic effect are the main contributing factors to KR-substrate binding, as shown in MMPBSA results where $\Delta G_{vdw}$ and $\Delta G_{total}$ consistently show virtually identical patterns for each KR-ligand pair (Figure 5 and Figure 6 and Figure S6). This implies that although the electrostatic interactions between negatively charged phosphopantetheine and positively charged patches play certain role in stabilizing KR-substrate interactions, van der Waals interaction and hydrophobic effects between the uncharged polyketide moiety and binding pockets are still the dominant contributors to KR-ligands binding specificity.

## 3. Discussion

### 3.1. Sequence Analysis of ActKR and HedKR

The antibiotic actinorhodin is synthesized by a type II PKS, which generates 16 carbon intermediate (octaketide) that is reduced by *Act*KR at the C9 carbonyl group. *Act*KR has been shown to be highly specific in reducing octaketides over other ketide lengths, with much-reduced activity for a hexaketide (12 carbons) [14]. In contrast, *Hed*KR, involved in hedamycin synthesis, is much more promiscuous, reducing tetra-, octa-, nona-, undeca- and dodeca-ketides (8, 16, 18, 22, 24 carbons). It remains unknown what leads to the observed difference in substrate specificity between *Act*KR and *Hed*KR. Our previous studies identified four important aspects guiding *Act*KR substrate specificity [15]: (1) An Arg-rich surface patch responsible for ACP and phospho-pantetheine binding, (2) “gate” residues controlling substrate access to the active site, (3) “steering” residues that guide the pantetheine-bound polyketide towards the active site, and (4) cyclizing residues responsible for first ring cyclization. However, sequence alignment shows that some of the identified residues are conserved between *Act*KR and *Hed*KR. For instance, V151 and V154, which belong to the “steering” residues group, and Y202, which may stabilize the flexible α6–α7 helices via π-π interactions with W206, are all conserved, (Figure S1) indicating those residues are not the reasons why these two proteins have different substrate specificity. A close inspection of the sequence alignment results revealed that H153 is proximal to V151 and V154, and H201 is proximal to Y202, which are not conserved between these two proteins. Thus, the H153Y/H201G DM-*Act*KR was generated with the hope that the double mutation will increase the promiscuity of *Act*KR so that it can accept polyketides of lengths other than 16 carbon.

DM-*Act*KR was co-crystalized with pantetheinylated tetraketide and phosphopantetheinylated octaketide isoxazole mimics. Well-defined electron density of both mimics can be observed inside the DM-*Act*KR active site pocket. As expected, DM-*Act*KR can accept both long (16 carbon) and short (eight carbon) polyketides. We have previously proposed that flexible and less conserved α6–α7 helices are important for substrate recognition, [15] the double mutant may have removed the hydrogen bonding interactions at the substrate pocket entrance that could trap shorter polyketides outside the active site.

### 3.2. Structures of ACP-Polyketide-KR Complexes Are Still Needed

The acyl carrier protein (ACP) is a critical component in both fatty acid and polyketide biosynthesis. Throughout synthesis, the growing product chains are bound as thiol esters at the distal thiol of the ACP’s phosphopantetheine moiety and are thus transported to required protein for each synthetic step [26]. We note that we performed all the MD simulations in the absence of ACP which would be present in vivo. Previous studies have postulated that the positively charged front-patch that promotes complementary interactions with both helix II of the ACP and the phosphopantetheine [27,28,29]. The co-crystal structure of DM-*Act*KR-(m-tet-p) first identified the back-patch, which is also positively charged. However, it is noticeable that ketoreductases for type II PKSs tend to exist in the form of tetramer; therefore, only the front-patches are exposed to the outer surface, while the back-patches are buried inside the interface between attaching monomers, which may not have enough space for ACP binding (Figure S8). Thus, the back-patch may only function as ligand binding patch in experimental conditions where the ligands are not attached to ACPs. Therefore, the structures of ACP-polyketide-KR complexes are still urgently needed to reveal the natural mechanism of KR-polyketide binding in detail.

## 4. Materials and Methods

### 4.1. Molecular Docking

Molecular docking analysis was conducted using three DM-*Act*KR crystal structures as the docking templates: the previously solved DM-*Act*KR co-crystal structure bound with the octaketide-phosphopantetheine mimic, the DM-*Act*KR co-crystal structure bound with the tetraketide-pantetheine mimic, and the apo structure of DM-*Act*KR mutated in silico. Modeller [30] was used to generate the apo structure of DM-*Act*KR mutant in silico from a previously solved apo structure of WT-*Act*KR (PDB ID: 1X7H) [9]. The models were prepared by selecting the monomer subunits that contain the substrate mimic in each tetramer structure and manually deleting the mimic coordinates. In order to enhance the exhaustiveness and specificity of the docking analysis by limiting the amount of degrees of freedom on conformation space, a fragment containing the phosphate group and part of the pantetheine (Figure S2) was designed as the docking ligand. AutoDock Vina [31] was used with the default scoring function. The dimensions of the search box were 25.04 × 25.74 × 30.82 Å, centered to include the entire model in each run to avoid biasing binding position. Search exhaustiveness was set to 10,000 to sufficiently sample ligand binding modes. The first 200 binding modes with the highest scores were visually assessed using UCSF Chimera [32].

### 4.2. MD Preparations

To prepare each KR-ligand complex for MD simulation, two previously solved DM-*Act*KR co-crystal structures (manuscript in preparation) were used as templates to place ligands into the binding pocket by alignment. All ketoreductases models were prepared as tetramers to match their native multimeric state. To parameterize small molecules including the 4 co-enzyme NADPHs associated with each KR monomer and each ligand, the AM1-BCC charging method, derived from the *antechamber* program, was used, [33,34] and the *parmchk2* program was used to prepare the missing parameters. Topology and coordinate files for the KR-ligand complexes were prepared using the *tleap* module. Following parametrization, the KR-ligand complexes were solvated in an octahedral box of TIP3P water molecules with thickness extending 10 Å from the protein surface [35] and complexes were neutralized by adding sodium ions.

### 4.3. MD Simulations

All MD simulations were performed using the *pmemd.cuda* program from the *Amber 18* software suite [36,37]. A 10 Å cutoff was used for nonbonded interactions and short-range electrostatic corrections. Long-range electrostatic interactions were handled by the particle mesh Ewald (PME) method [38,39]. The hydrogen atom bond lengths were fixed with the SHAKE algorithm [40,41]. Minimization was performed in two steps to relieve any possible atomic overlaps. The first step involved relaxing only water molecules, while the second step minimized the whole system. Langevin dynamics with a 1 ps^−1^ collision frequency were used to gradually increase system temperature from 0 to 300 K over 200 ps [42]. Prior to production stage simulations, the system was equilibrated for 100 ns under constant pressure and temperature (NPT) to adjust the system density. Finally, 100 ns production simulations without any restraint were performed under constant volume and temperature (NVT) conditions. Each simulation was repeated three times with a different random seed, starting from identical minimized structures. A 2 fs integration time step was utilized with structural snapshots extracted every 1 ns.

### 4.4. MD Analysis

All simulation trajectories were visualized using the software VMD [43]. The stability score (SS) was developed to determine how stable a receptor–ligand interaction is during a simulation. The native atom pairs are defined as the heavy atom pairs that are within the distance of 7 Å in the initial frame. In any subsequent frame, the stability score is the fraction of the amount of these pairs that remain within 7 Å of each other, with 1 indicating that the ligand position closely matches the initial frame, and 0 indicating ligand exit from its original binding site. Thus, SS of the first frame is always 1 for each trajectory, and SS is less than or equal to 1 for subsequent frames. RMSD and Stability Score SS of each simulation trajectories were calculated using the *cpptraj* module in *AmberTools18* [44].

MMPBSA calculations [21,22,23,24,25] were conducted on the last 100 ns of each MD trajectory (frame interval is 1 ns) using the *MMPBSA.py* module in *AmberTools18*. The ionic strength was set at 0.100 M to reflect the sodium ions originally present in the simulations. Because KR active sites are highly charged, the internal dielectric constant was set to 4, which is suitable for charged receptor–ligand systems [45]. Due to time and computational resource limitations, the normal-mode-based entropy corrections to these values were not calculated as they do not improve agreement with measured affinities.

All statistical analyses were conducted by using *R* statistical packages.

## 5. Conclusions

The regiospecific reduction of a single carbonyl group to a hydroxyl group catalyzed by ketoreductase (KR) is an essential step of reducing-type polyketide synthesis [20]. Several important ketoreductase structures from reducing type II PKS have been determined, including actinorhodin (*Act*KR) [9] and hedamycin (*Hed*KR) [15]. However, the mechanism of the ketoreductase–substrate interaction is still not well-known due to the fact that the poly-β-ketone intermediates which ketoreductases act on are highly reactive and prone to spontaneous cyclization, making them challenging to isolate. The primary solutions to overcome inherent reactivity to study the ketoreductase–substrate interaction include substrate mimics and computer simulation.

Using these two approaches, we made five important observations on KR-substrate binding on co-crystal structures. First, docking results show that the previously proposed back-patch and front-patch residues are two major sites for substrate binding in *Ac*tKR, regardless of conformation. Second, polyketide length is the key determinant for which of the two sites a substrate will bind to in a KR. Third, H153 and H201 of *Act*KR are key gating residues for substrate chain length specificity, and the mutation of these two residues towards corresponding residues in *Hed*KR increased the binding affinity of *Act*KR towards polyketide substrates with different chain lengths. Fourth, pantetheine or phosphopantetheine are essential for substrate binding, and the binding affinity of most ligands with KR increased significantly in the presence of a phosphate group on the ligand. Finally, packing/hydrophobic effects are the main contributing factors to KR-substrates binding stability.

Understanding the detailed molecular basis for KR-substrate binding is crucial for rationally engineering type II PKS systems. The molecular features identified in this work will serve as protein engineering targets for rational control of KR specificity to produce new polyketides with pharmaceutical potential.

## Figures and Tables

**Table 1:** Molecular dynamics (MD) simulation round 1, including 24 DM-*Act*KR-ligand complexes prepared through structure alignment using DM-*Act*KR-(m-oct-pp) and DM-*Act*KR-(m-tet-p) co-crystal structures as templates.

Aligned to DM-*Act*KR Front-Patch ^[a][b]^			Aligned to DM-*Act*KR Back-Patch ^[a][b]^		
m-tet	m-tet-p	m-tet-pp	m-tet	m-tet-p ^[c]^	m-tet-pp
tet	tet-p	tet-pp	tet	tet-p	tet-pp
m-oct	m-oct-p	m-oct-pp ^[c]^	m-oct	m-oct-p	m-oct-pp
oct	oct-p	oct-pp	oct	oct-p	oct-pp

^[a]^ Each DM-*Act*KR-ligand pair were simulated in triplicate. ^[b]^ Ligand nomenclatures explained. Prefix: “m” means isoxazole mimic, without “m” means natural structure; Body: “tet” means tetraketide, “oct” means octaketide; Suffix: “p” means (unphosphorylated) pantetheine, “pp” means phosphopantetheine, and without suffix means the ligand only has polyketide moiety. ^[c]^ DM-*Act*KR-(m-oct-pp) (ligand binds to front-patch) and DM-*Act*KR-(m-tet-p) (ligand binds to back-patch) are experimental structures.

**Table 2:** MD Simulation Round 2, including 4 WT-*Act*KR-ligand complexes and 4 WT-*Hed*KR-ligand complexes prepared through structure alignment, using DM-*Act*KR-(m-oct-pp) and DM-*Act*KR-(m-tet-p) co-crystal structures as templates.

**Aligned to WT-*Act*KR Front-Patch ^[a][b]^**		**Aligned to WT-*Act*KR Back-Patch ^[a][b]^**	
oct-pp	oct-p	tet-pp	tet-p
**Aligned to WT-*Hed*KR front-patch**		**Aligned to WT-*Hed*KR back-patch**	
oct-pp	oct-p	tet-pp	tet-p

^[a]^ Each DM-*Act*KR-ligand pair were simulated in triplicate. ^[b]^ Ligand nomenclatures are the same as Table 1.

## Supplementary Materials

Supplementary materials can be found at https://www.mdpi.com/1422-0067/21/20/7562/s1, Figure S1: Sequence alignment among various type II PKS KRs, Figure S2: The fragment used in molecular docking, Figure S3: The RMSD plots and stability score $SS_{i}$ plots of each simulation trajectories, Figure S4: Front view of DM-ActKR displaying the relative positions of front patch, back patch and catalytic residues, Figure S5: Hanging chain effect comparison between DM-ActKR-tet-pp binding and DM-ActKR-tet-p binding, Figure S6: MMPBSA comparison of octaketides and tetraketides bound to DM-ActKR, WT-ActKR and WT-HedKR, Figure S7: Framewise Pearson correlation test between $\Delta G_{total}$ with $\Delta G_{vdw}$ or $\Delta G_{ele}$ of each KR-ligand pair, Figure S8: The position of the front patch and back patch in *Act*KR tetramer.
