# Supplementary material for: Molecular Basis for Polyketide Ketoreductase–Substrate Interactions
Source: Int J Mol Sci. 2020 Oct 13;21(20):7562. doi: 10.3390/ijms21207562 (PMC7588967; doi:10.3390/ijms21207562)
Supplement: Supplementary file 1 [file ijms-21-07562-s001.pdf]

International Journal of
Molecular Sciences

MDPI

Supplementary matetials

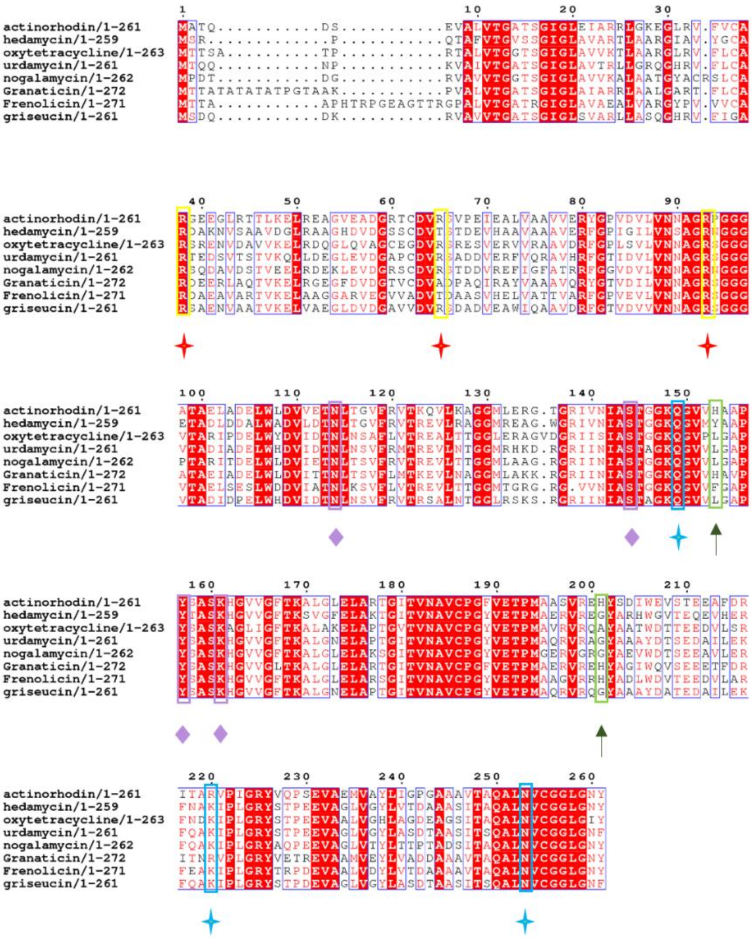

Figure S1. Sequence alignment among various type II PKS KRs. Sequences included actinorhodin, hedamycin, oxytetracycline, urdamycin, nogalamycin, granaticin, frenolicin, and griseucin KRs. Key: Red stars, front-patch residues; Cyan stars, back-patch residues; Purple diamonds, catalytic residues; Green arrows, proposed chain length filter residues (double mutation targets on WT-*Act*KR).

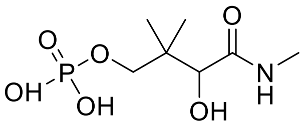

3-hydroxy-2,2-dimethyl-4-(methylamino)-4-oxobutyl dihydrogen phosphate

Figure S2. The phosphopantetheine fragment used in molecular docking.

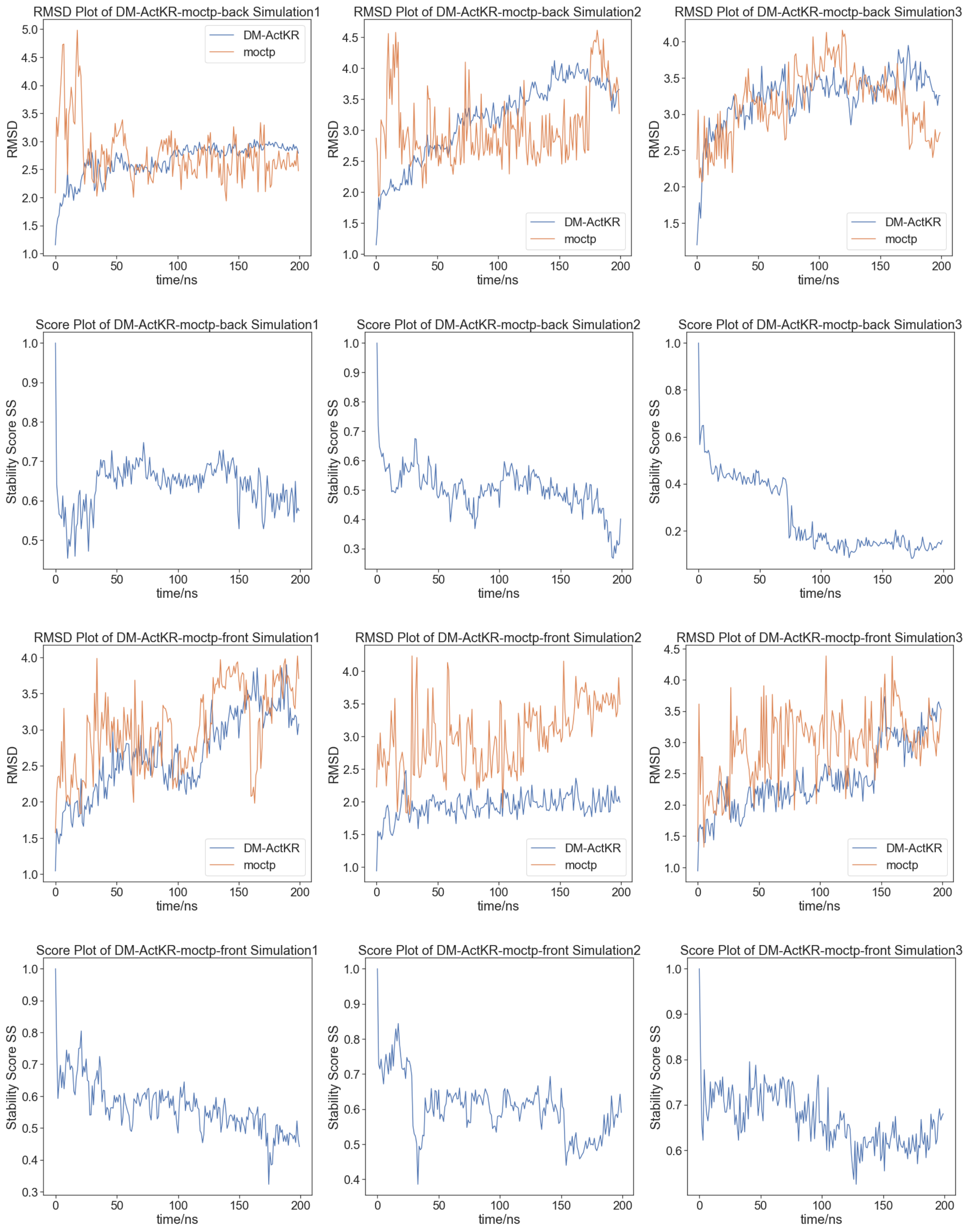
RMSD Plot of DM-ActKR-moctp-back Simulation1
RMSD Plot of DM-ActKR-moctp-back Simulation2
RMSD Plot of DM-ActKR-moctp-back Simulation3
Score Plot of DM-ActKR-moctp-back Simulation1
Score Plot of DM-ActKR-moctp-back Simulation2
Score Plot of DM-ActKR-moctp-back Simulation3
RMSD Plot of DM-ActKR-moctp-front Simulation1
RMSD Plot of DM-ActKR-moctp-front Simulation2
RMSD Plot of DM-ActKR-moctp-front Simulation3
Score Plot of DM-ActKR-moctp-front Simulation1
Score Plot of DM-ActKR-moctp-front Simulation2
Score Plot of DM-ActKR-moctp-front Simulation3
RMSD
Stability Score SS
time/ns
DM-ActKR
moctp

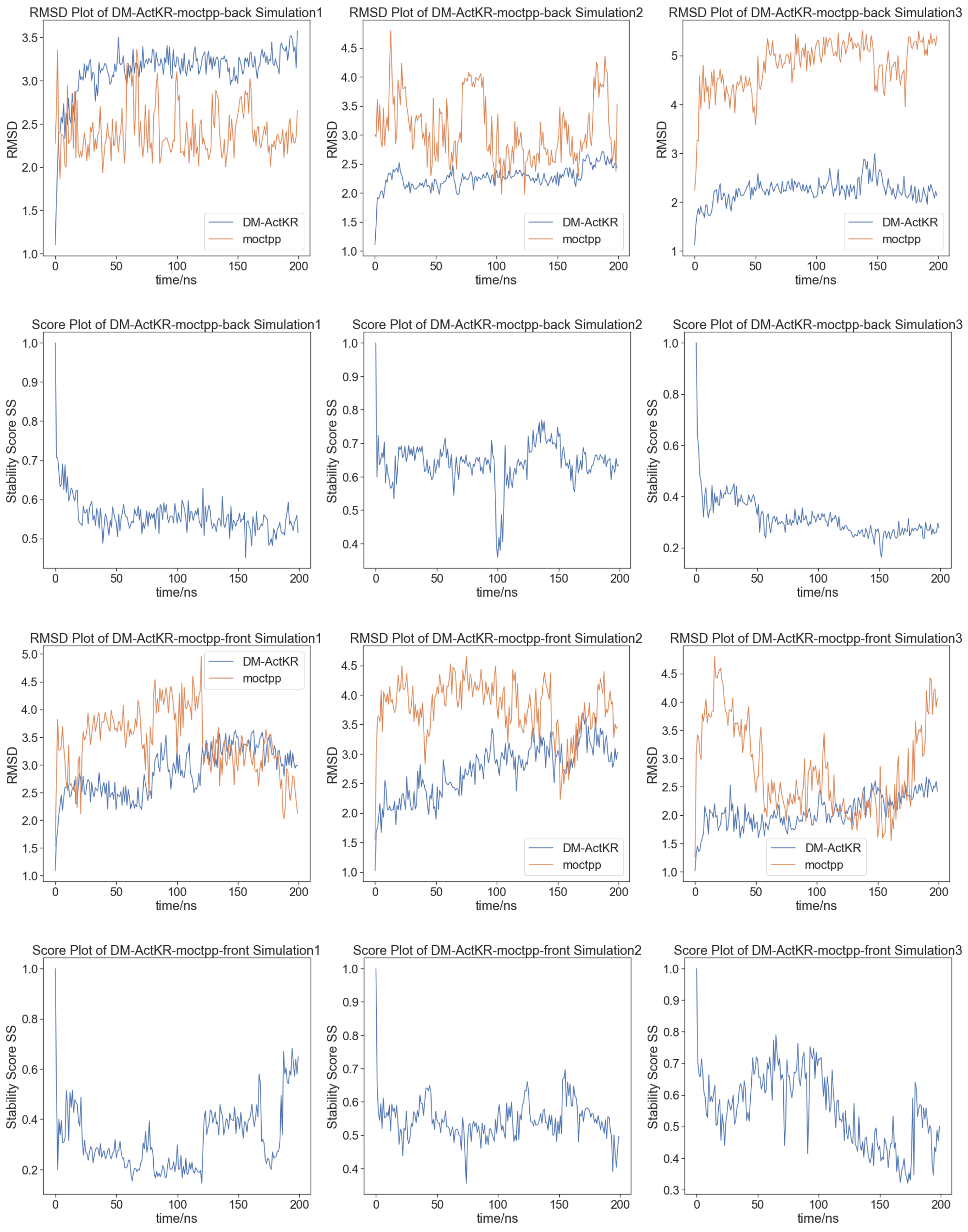
RMSD Plot of DM-ActKR-moctpp-back Simulation1
RMSD Plot of DM-ActKR-moctpp-back Simulation2
RMSD Plot of DM-ActKR-moctpp-back Simulation3
Score Plot of DM-ActKR-moctpp-back Simulation1
Score Plot of DM-ActKR-moctpp-back Simulation2
Score Plot of DM-ActKR-moctpp-back Simulation3
RMSD Plot of DM-ActKR-moctpp-front Simulation1
RMSD Plot of DM-ActKR-moctpp-front Simulation2
RMSD Plot of DM-ActKR-moctpp-front Simulation3
Score Plot of DM-ActKR-moctpp-front Simulation1
Score Plot of DM-ActKR-moctpp-front Simulation2
Score Plot of DM-ActKR-moctpp-front Simulation3
RMSD
Stability Score SS
time/ns
DM-ActKR
moctpp

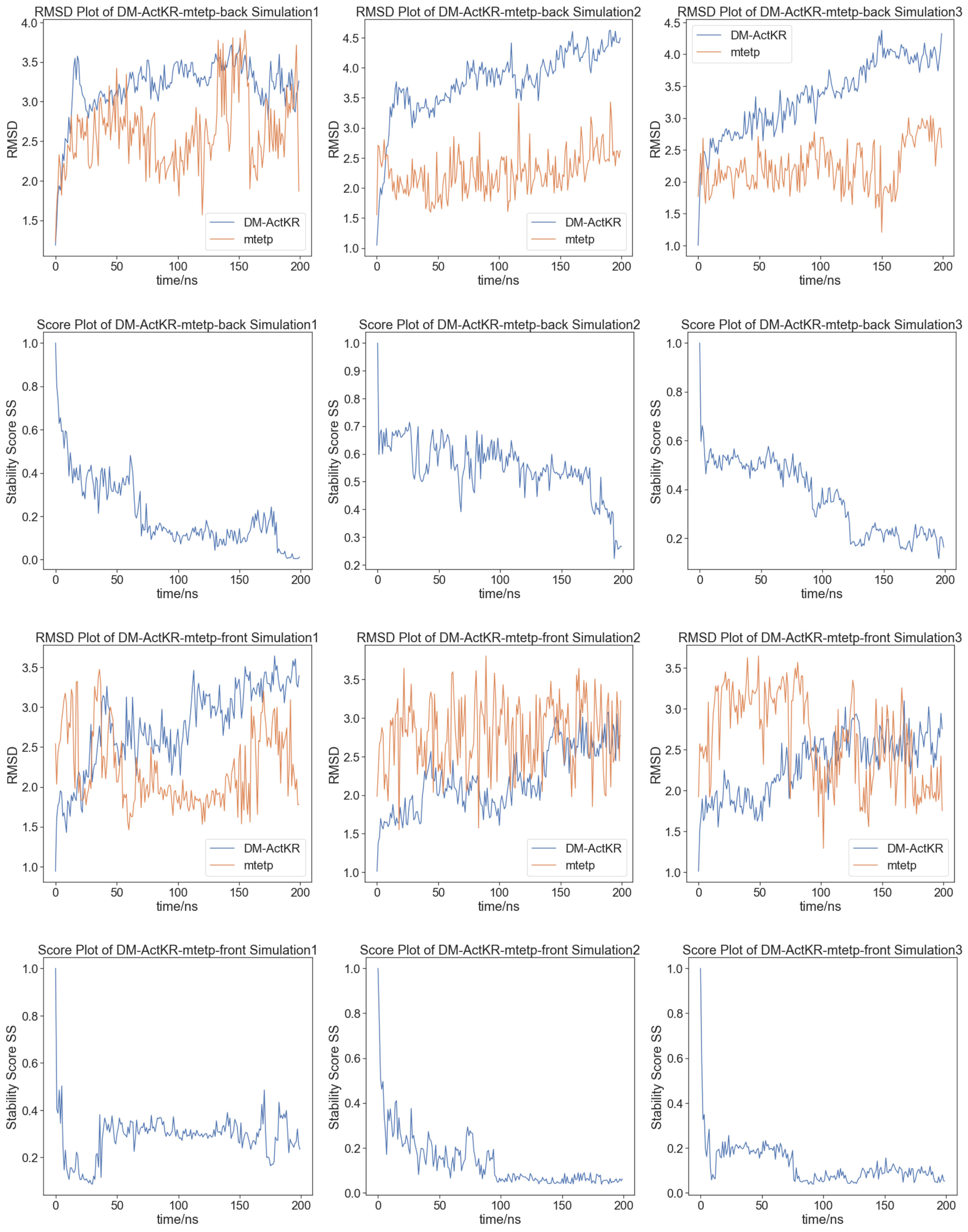
RMSD Plot of DM-ActKR-mtetp-back Simulation1
RMSD Plot of DM-ActKR-mtetp-back Simulation2
RMSD Plot of DM-ActKR-mtetp-back Simulation3
Score Plot of DM-ActKR-mtetp-back Simulation1
Score Plot of DM-ActKR-mtetp-back Simulation2
Score Plot of DM-ActKR-mtetp-back Simulation3
RMSD Plot of DM-ActKR-mtetp-front Simulation1
RMSD Plot of DM-ActKR-mtetp-front Simulation2
RMSD Plot of DM-ActKR-mtetp-front Simulation3
Score Plot of DM-ActKR-mtetp-front Simulation1
Score Plot of DM-ActKR-mtetp-front Simulation2
Score Plot of DM-ActKR-mtetp-front Simulation3
RMSD
Stability Score SS
time/ns
DM-ActKR
mtetp

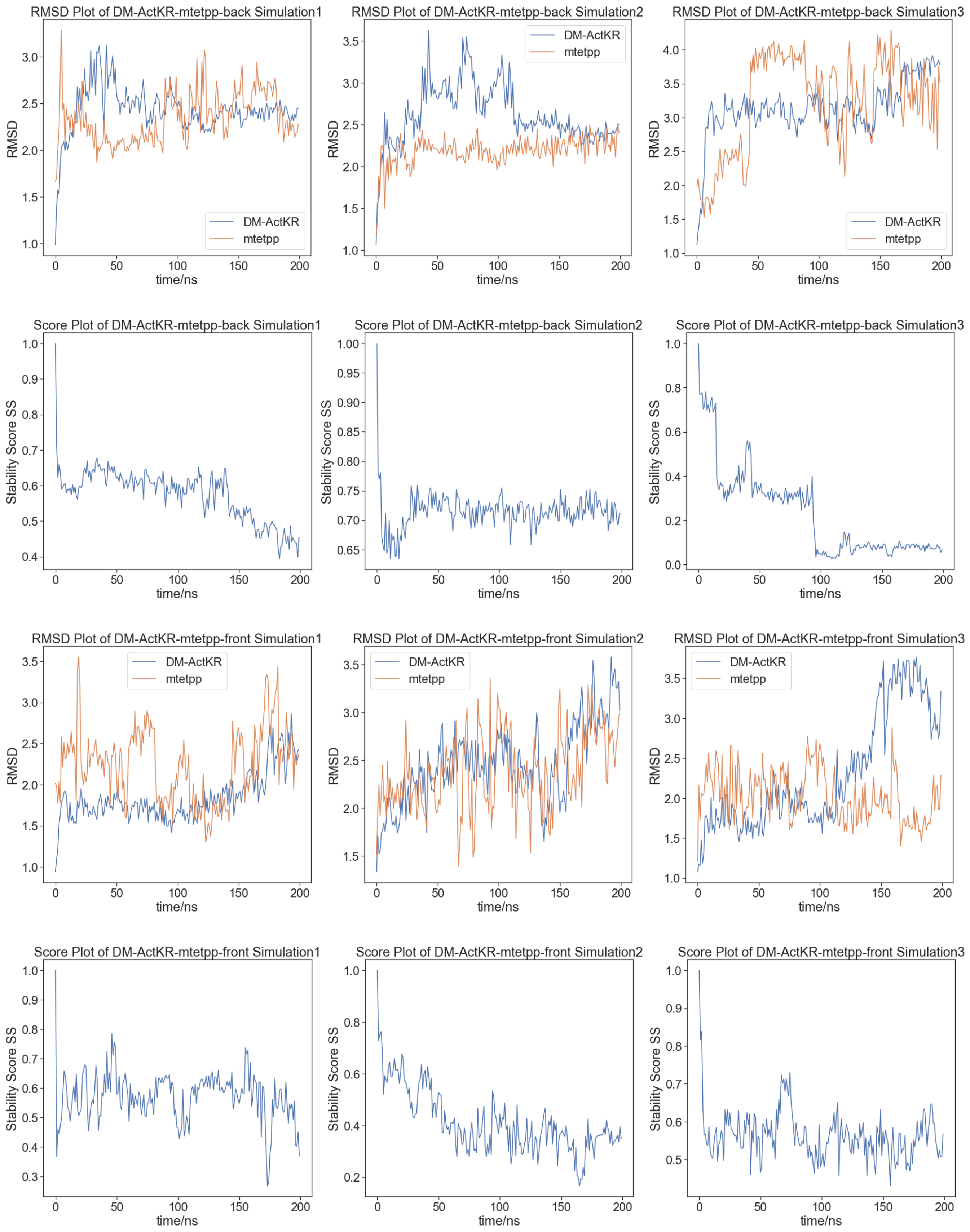
RMSD Plot of DM-ActKR-mtetpp-back Simulation1
RMSD Plot of DM-ActKR-mtetpp-back Simulation2
RMSD Plot of DM-ActKR-mtetpp-back Simulation3
Score Plot of DM-ActKR-mtetpp-back Simulation1
Score Plot of DM-ActKR-mtetpp-back Simulation2
Score Plot of DM-ActKR-mtetpp-back Simulation3
RMSD Plot of DM-ActKR-mtetpp-front Simulation1
RMSD Plot of DM-ActKR-mtetpp-front Simulation2
RMSD Plot of DM-ActKR-mtetpp-front Simulation3
Score Plot of DM-ActKR-mtetpp-front Simulation1
Score Plot of DM-ActKR-mtetpp-front Simulation2
Score Plot of DM-ActKR-mtetpp-front Simulation3
RMSD
Stability Score SS
time/ns
DM-ActKR
mtetpp

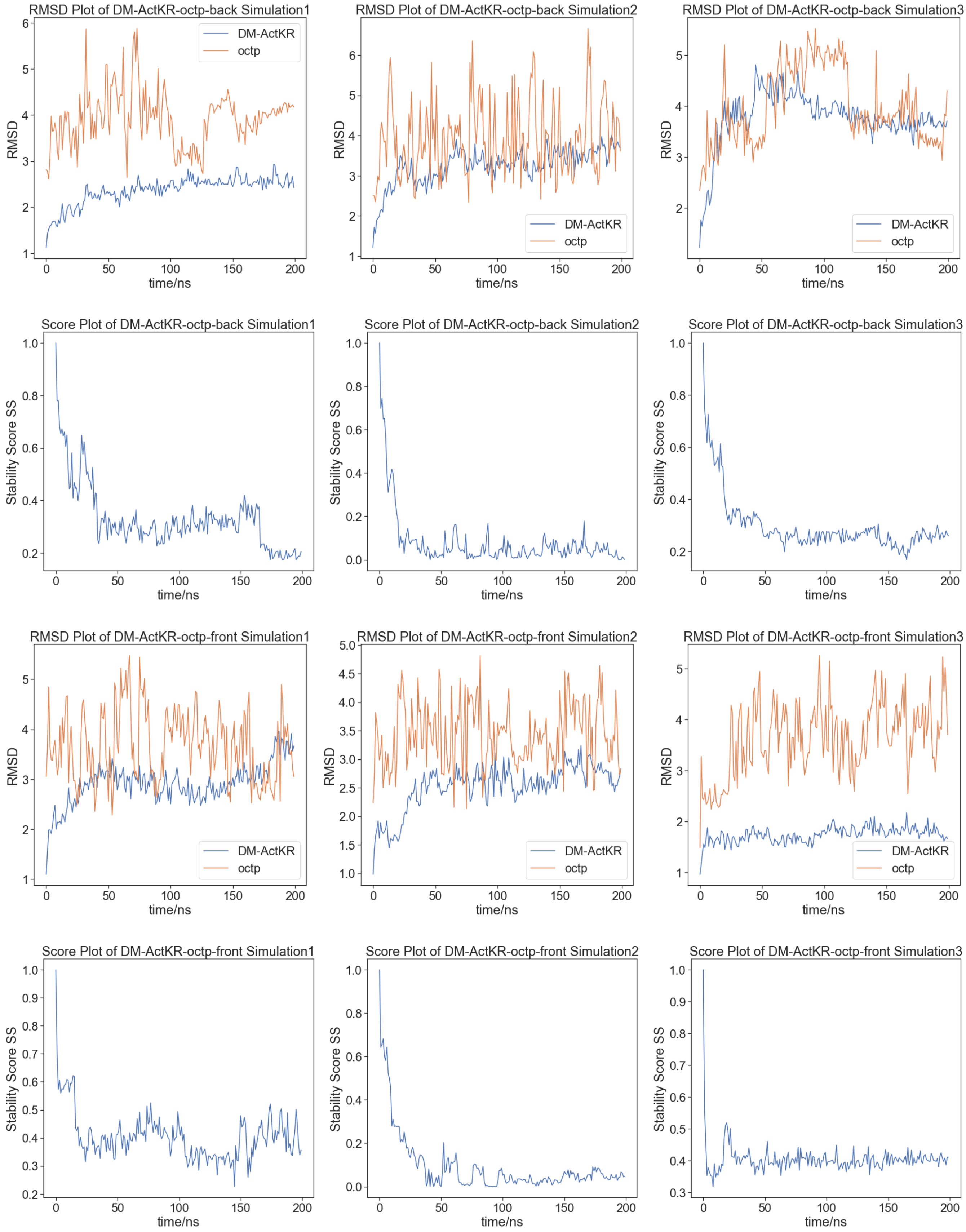
RMSD Plot of DM-ActKR-octp-back Simulation1
RMSD Plot of DM-ActKR-octp-back Simulation2
RMSD Plot of DM-ActKR-octp-back Simulation3
Score Plot of DM-ActKR-octp-back Simulation1
Score Plot of DM-ActKR-octp-back Simulation2
Score Plot of DM-ActKR-octp-back Simulation3
RMSD Plot of DM-ActKR-octp-front Simulation1
RMSD Plot of DM-ActKR-octp-front Simulation2
RMSD Plot of DM-ActKR-octp-front Simulation3
Score Plot of DM-ActKR-octp-front Simulation1
Score Plot of DM-ActKR-octp-front Simulation2
Score Plot of DM-ActKR-octp-front Simulation3
DM-ActKR
octp
RMSD
Stability Score SS
time/ns

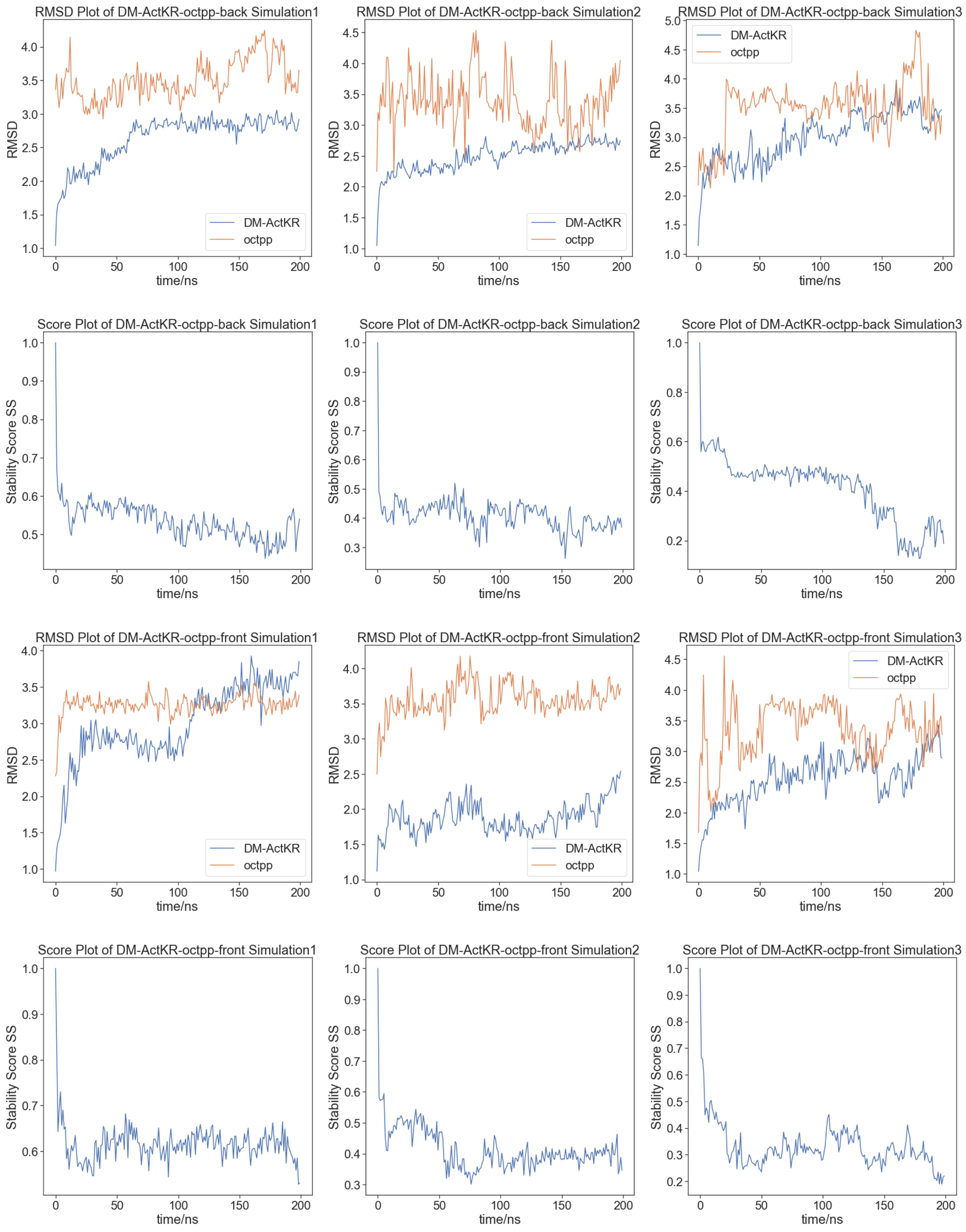
RMSD Plot of DM-ActKR-octpp-back Simulation1
RMSD Plot of DM-ActKR-octpp-back Simulation2
RMSD Plot of DM-ActKR-octpp-back Simulation3
Score Plot of DM-ActKR-octpp-back Simulation1
Score Plot of DM-ActKR-octpp-back Simulation2
Score Plot of DM-ActKR-octpp-back Simulation3
RMSD Plot of DM-ActKR-octpp-front Simulation1
RMSD Plot of DM-ActKR-octpp-front Simulation2
RMSD Plot of DM-ActKR-octpp-front Simulation3
Score Plot of DM-ActKR-octpp-front Simulation1
Score Plot of DM-ActKR-octpp-front Simulation2
Score Plot of DM-ActKR-octpp-front Simulation3
RMSD
Stability Score SS
time/ns
DM-ActKR
octpp

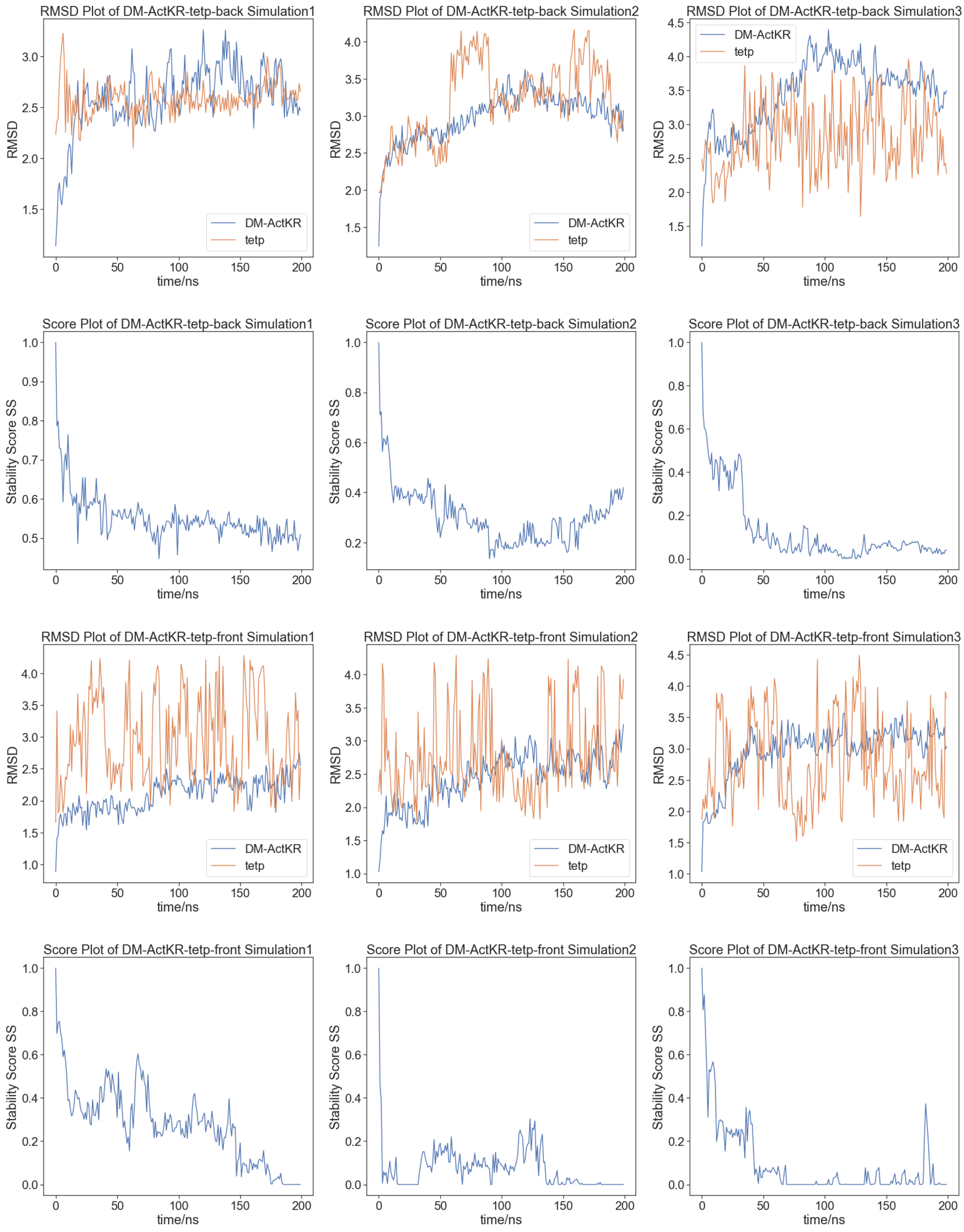
RMSD Plot of DM-ActKR-tetp-back Simulation1
RMSD Plot of DM-ActKR-tetp-back Simulation2
RMSD Plot of DM-ActKR-tetp-back Simulation3
Score Plot of DM-ActKR-tetp-back Simulation1
Score Plot of DM-ActKR-tetp-back Simulation2
Score Plot of DM-ActKR-tetp-back Simulation3
RMSD Plot of DM-ActKR-tetp-front Simulation1
RMSD Plot of DM-ActKR-tetp-front Simulation2
RMSD Plot of DM-ActKR-tetp-front Simulation3
Score Plot of DM-ActKR-tetp-front Simulation1
Score Plot of DM-ActKR-tetp-front Simulation2
Score Plot of DM-ActKR-tetp-front Simulation3
RMSD
Stability Score SS
time/ns
DM-ActKR
tetp

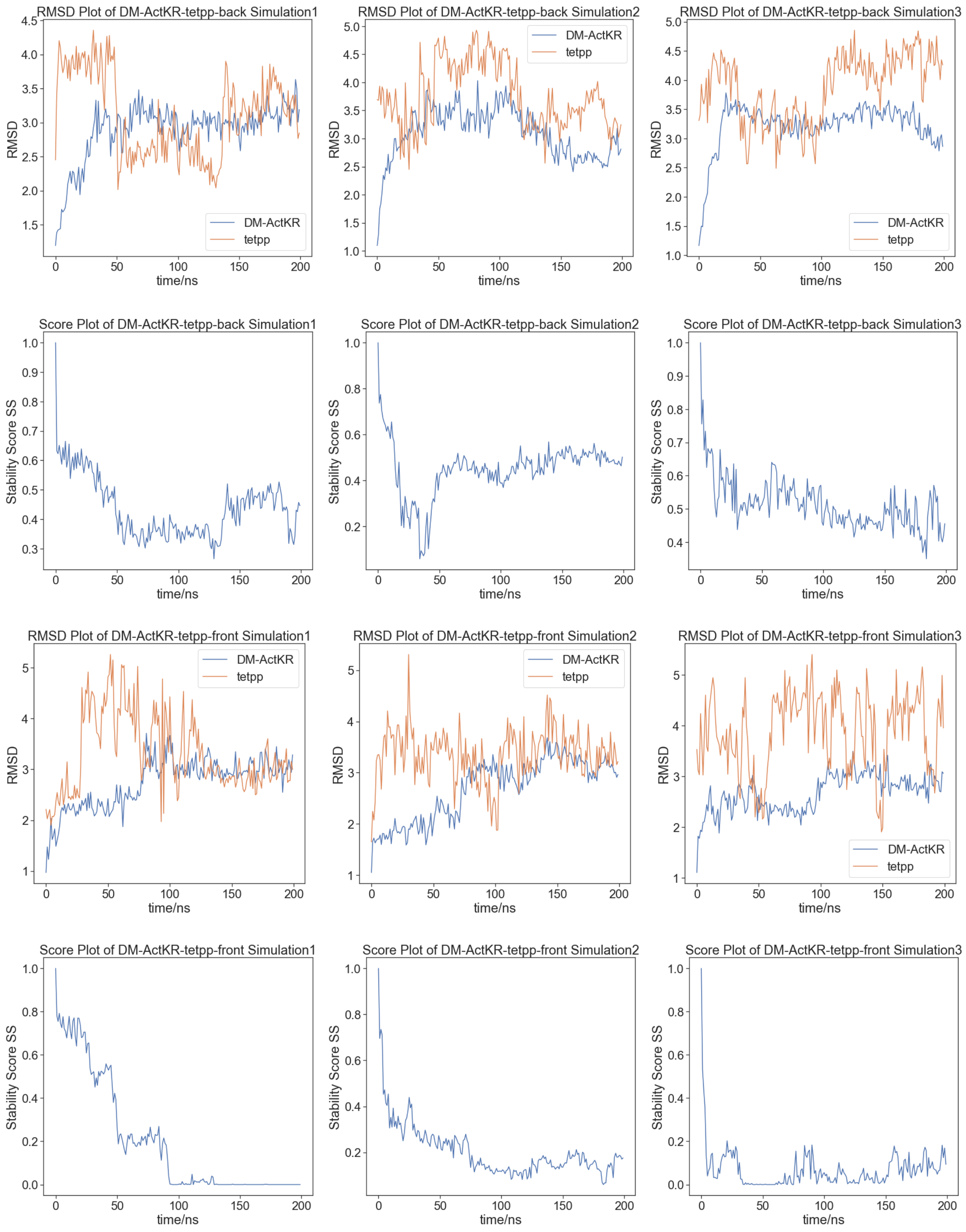
RMSD Plot of DM-ActKR-tetpp-back Simulation1
RMSD Plot of DM-ActKR-tetpp-back Simulation2
RMSD Plot of DM-ActKR-tetpp-back Simulation3
Score Plot of DM-ActKR-tetpp-back Simulation1
Score Plot of DM-ActKR-tetpp-back Simulation2
Score Plot of DM-ActKR-tetpp-back Simulation3
RMSD Plot of DM-ActKR-tetpp-front Simulation1
RMSD Plot of DM-ActKR-tetpp-front Simulation2
RMSD Plot of DM-ActKR-tetpp-front Simulation3
Score Plot of DM-ActKR-tetpp-front Simulation1
Score Plot of DM-ActKR-tetpp-front Simulation2
Score Plot of DM-ActKR-tetpp-front Simulation3
DM-ActKR
tetpp
RMSD
Stability Score SS
time/ns

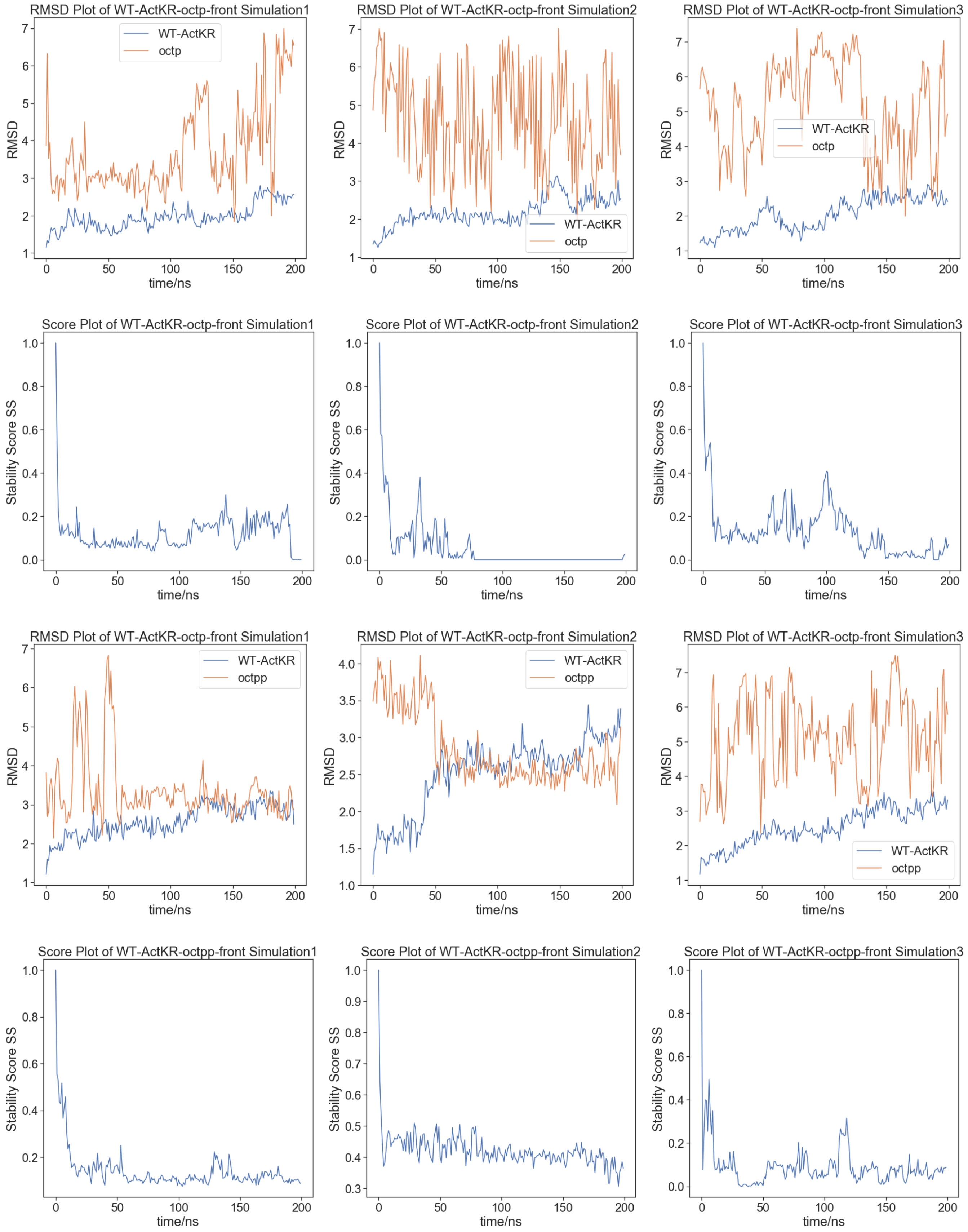
RMSD Plot of WT-ActKR-octp-front Simulation1
RMSD Plot of WT-ActKR-octp-front Simulation2
RMSD Plot of WT-ActKR-octp-front Simulation3
WT-ActKR
octp
RMSD
time/ns
Score Plot of WT-ActKR-octp-front Simulation1
Score Plot of WT-ActKR-octp-front Simulation2
Score Plot of WT-ActKR-octp-front Simulation3
Stability Score SS
time/ns
RMSD Plot of WT-ActKR-octp-front Simulation1
RMSD Plot of WT-ActKR-octp-front Simulation2
RMSD Plot of WT-ActKR-octp-front Simulation3
WT-ActKR
octpp
RMSD
time/ns
Score Plot of WT-ActKR-octpp-front Simulation1
Score Plot of WT-ActKR-octpp-front Simulation2
Score Plot of WT-ActKR-octpp-front Simulation3
Stability Score SS
time/ns

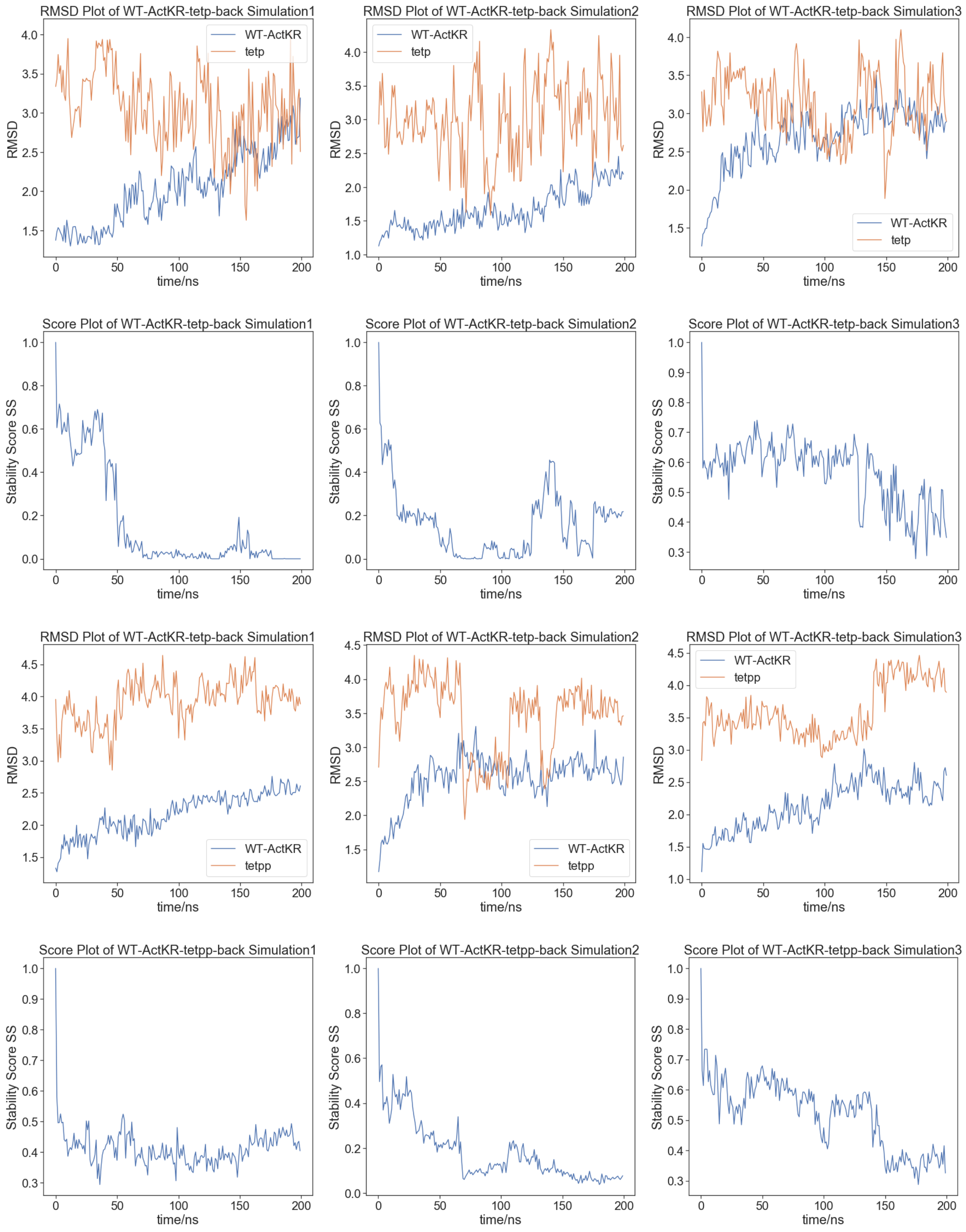
RMSD Plot of WT-ActKR-tetp-back Simulation1
RMSD Plot of WT-ActKR-tetp-back Simulation2
RMSD Plot of WT-ActKR-tetp-back Simulation3
WT-ActKR
tetp
RMSD
time/ns
Score Plot of WT-ActKR-tetp-back Simulation1
Score Plot of WT-ActKR-tetp-back Simulation2
Score Plot of WT-ActKR-tetp-back Simulation3
Stability Score SS
time/ns
RMSD Plot of WT-ActKR-tetp-back Simulation1
RMSD Plot of WT-ActKR-tetp-back Simulation2
RMSD Plot of WT-ActKR-tetp-back Simulation3
WT-ActKR
tetpp
RMSD
time/ns
Score Plot of WT-ActKR-tetpp-back Simulation1
Score Plot of WT-ActKR-tetpp-back Simulation2
Score Plot of WT-ActKR-tetpp-back Simulation3
Stability Score SS
time/ns

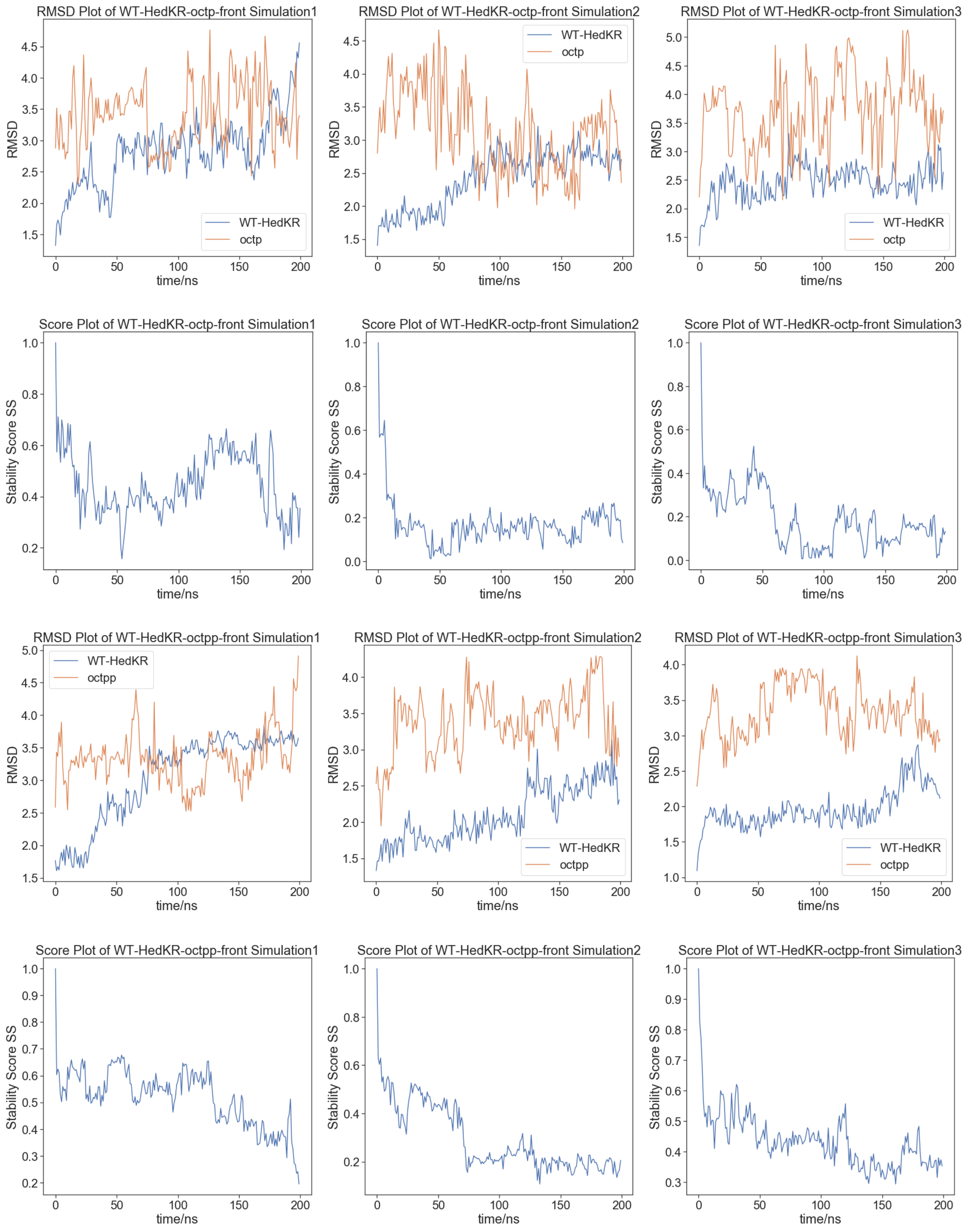
RMSD Plot of WT-HedKR-octp-front Simulation1
RMSD Plot of WT-HedKR-octp-front Simulation2
RMSD Plot of WT-HedKR-octp-front Simulation3
RMSD
time/ns
WT-HedKR
octp
Score Plot of WT-HedKR-octp-front Simulation1
Score Plot of WT-HedKR-octp-front Simulation2
Score Plot of WT-HedKR-octp-front Simulation3
Stability Score SS
time/ns
RMSD Plot of WT-HedKR-octpp-front Simulation1
RMSD Plot of WT-HedKR-octpp-front Simulation2
RMSD Plot of WT-HedKR-octpp-front Simulation3
RMSD
time/ns
WT-HedKR
octpp
Score Plot of WT-HedKR-octpp-front Simulation1
Score Plot of WT-HedKR-octpp-front Simulation2
Score Plot of WT-HedKR-octpp-front Simulation3
Stability Score SS
time/ns

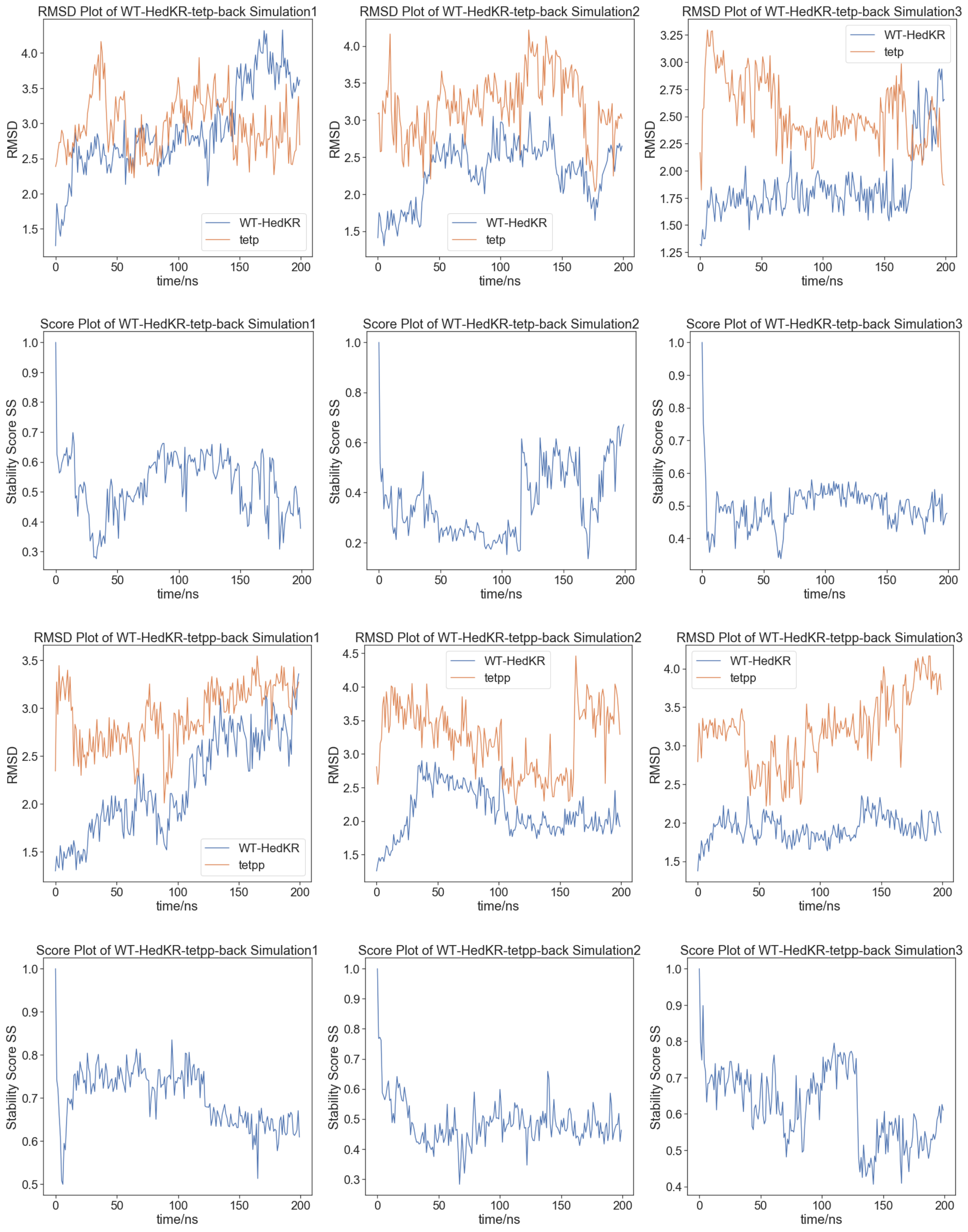

Figure S3. The RMSD and Stability Score SS_i plots of each simulation trajectory. The RMSD-simulation time plots have KR displayed in blue, ligands displayed in orange. The Stability Score SS_i-simulation time plots have Stability Score displayed in blue.

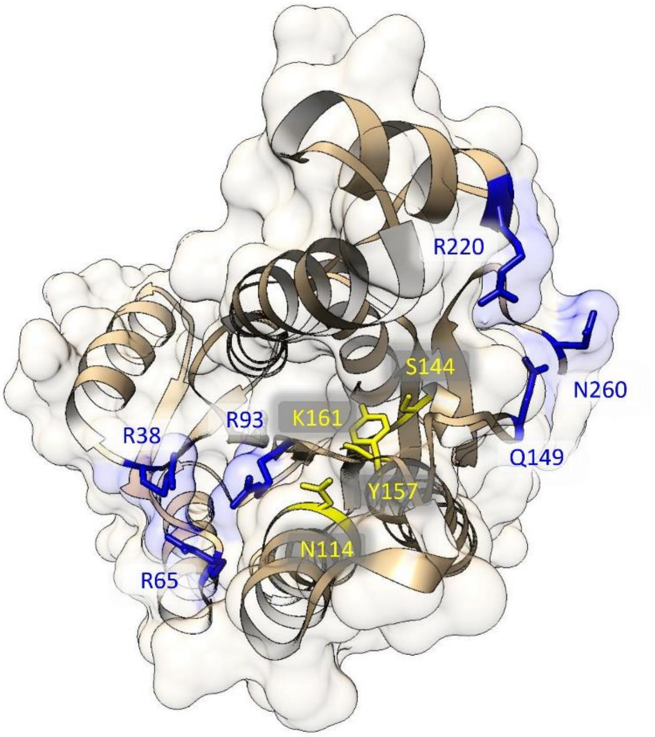

Figure S4. Front view of DM-*Act*KR displaying the relative positions of front patch, back patch and catalytic residues. The front-patch (R38, R65, R93) and the back-patch (Q149, R220, N260) form two opposite entrances of a long channel, in which the catalytic residues (N114, S144, Y157, K161) of active site are located at the center. Patch residues are displayed in blue and active site residues are displayed in yellow.

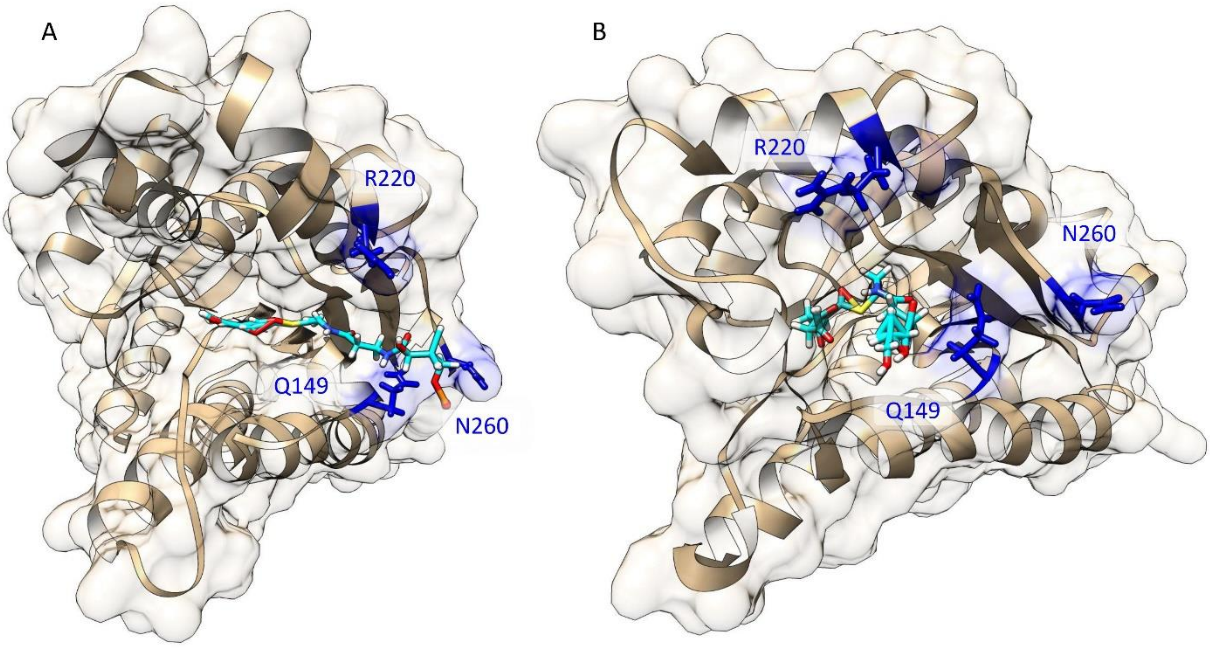

Figure S5. Hanging chain effect comparison between DM-*Act*KR-tet-pp binding and DM-*Act*KR-tet-p binding. Hanging chain effect is shown in DM-*Act*KR-tet-pp binding (A) but not in DM-*Act*KR-tet-p binding (A) Both figures show the average structure

of the last 100ns of the simulation trajectories. In (A), both ends of the ligand are constrained, and DM-*Act*KR is in open form. In (B), the pantetheine end of the ligand is not constrained, and DM-*Act*KR is in closed form.

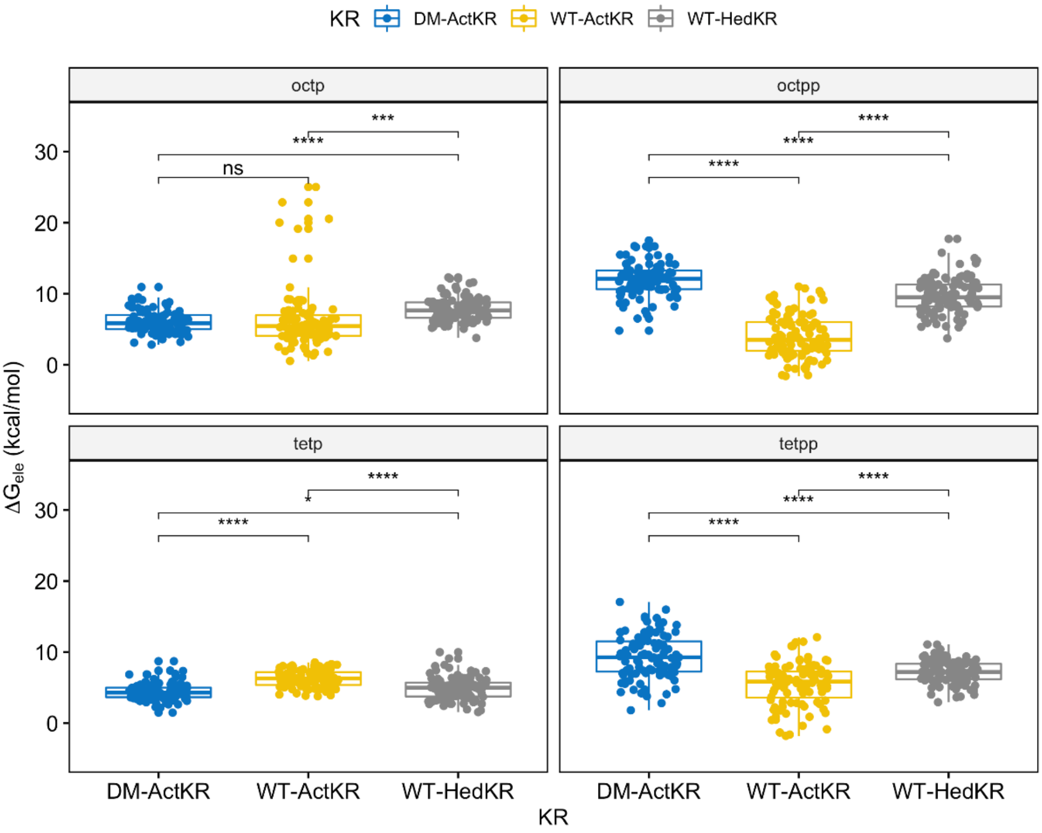

Figure S6. MMPBSA comparison of octaketides and tetraketides bound to DM-*Act*KR, WT-*Act*KR and WT-*Hed*KR. Each box plot shows the electrostatic energy ΔG_{ele} results.

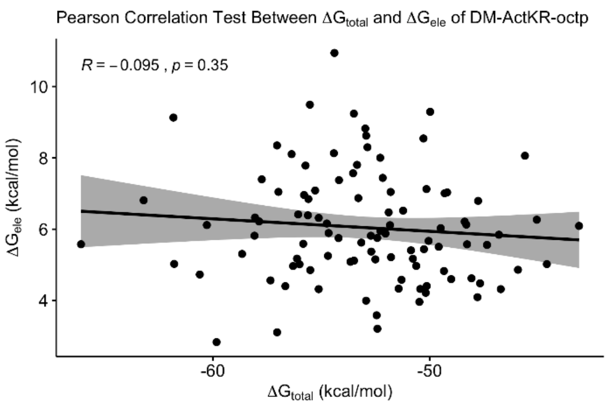
Pearson Correlation Test Between ΔG$_{total}$ and ΔG$_{ele}$ of DM-ActKR-octp
R = −0.095 , p = 0.35
ΔG$_{ele}$ (kcal/mol)
ΔG$_{total}$ (kcal/mol)

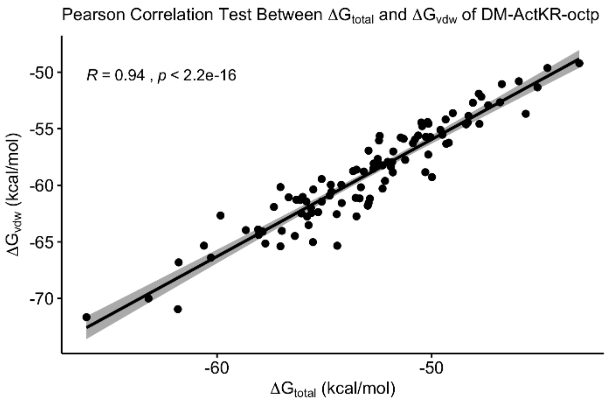
Pearson Correlation Test Between ΔG$_{total}$ and ΔG$_{vdw}$ of DM-ActKR-octp
R = 0.94 , p < 2.2e-16
ΔG$_{vdw}$ (kcal/mol)
ΔG$_{total}$ (kcal/mol)

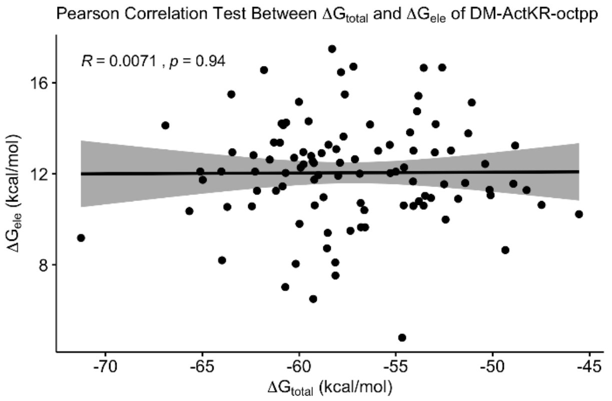
Pearson Correlation Test Between ΔG$_{total}$ and ΔG$_{ele}$ of DM-ActKR-octpp
R = 0.0071 , p = 0.94
ΔG$_{ele}$ (kcal/mol)
ΔG$_{total}$ (kcal/mol)

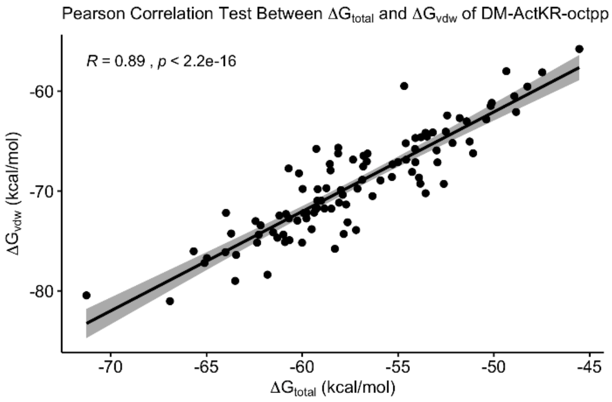
Pearson Correlation Test Between ΔG$_{total}$ and ΔG$_{vdw}$ of DM-ActKR-octpp
R = 0.89 , p < 2.2e-16
ΔG$_{vdw}$ (kcal/mol)
ΔG$_{total}$ (kcal/mol)

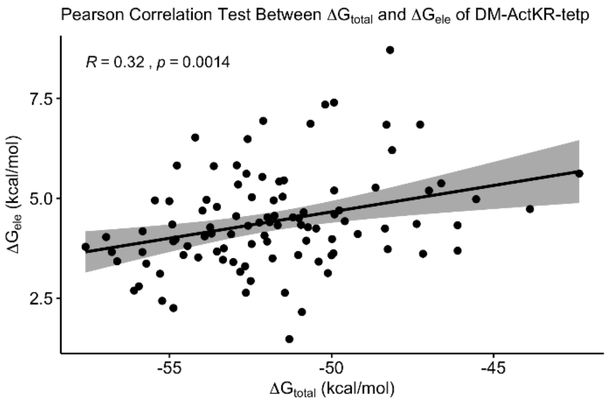
Pearson Correlation Test Between ΔG_{total} and ΔG_{ele} of DM-ActKR-tetp
R = 0.32 , *p* = 0.0014
ΔG_{ele} (kcal/mol)
ΔG_{total} (kcal/mol)
7.5
5.0
2.5
-55
-50
-45

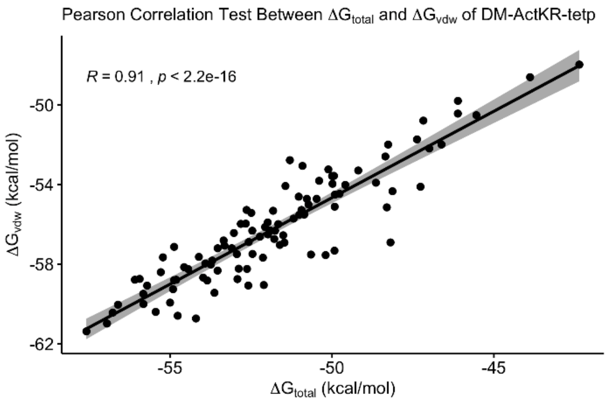
Pearson Correlation Test Between ΔG_{total} and ΔG_{vdw} of DM-ActKR-tetp
R = 0.91 , *p* < 2.2e-16
ΔG_{vdw} (kcal/mol)
ΔG_{total} (kcal/mol)
-50
-54
-58
-62
-55
-50
-45

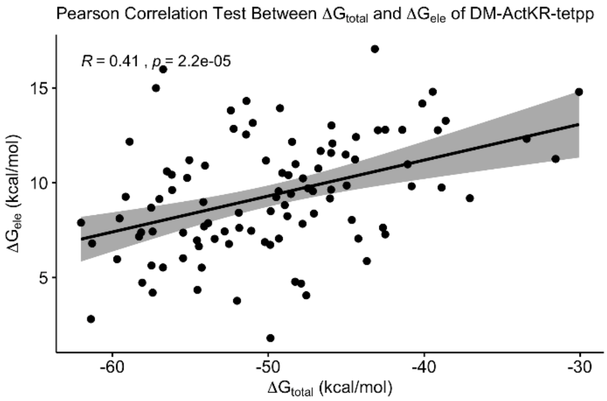
Pearson Correlation Test Between ΔG_{total} and ΔG_{ele} of DM-ActKR-tetpp
R = 0.41 , *p* = 2.2e-05
ΔG_{ele} (kcal/mol)
ΔG_{total} (kcal/mol)
15
10
5
-60
-50
-40
-30

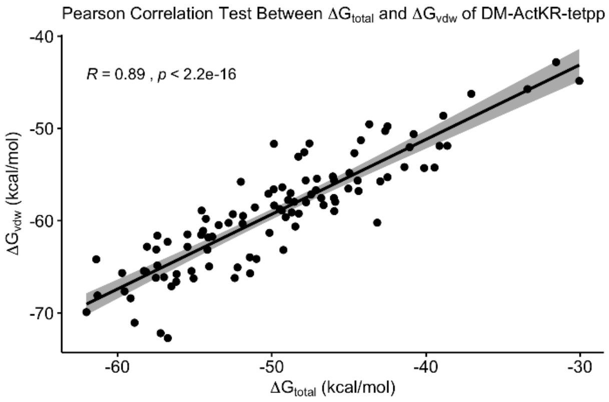
Pearson Correlation Test Between ΔG_{total} and ΔG_{vdw} of DM-ActKR-tetpp
R = 0.89 , *p* < 2.2e-16
ΔG_{vdw} (kcal/mol)
ΔG_{total} (kcal/mol)
-40
-50
-60
-70
-60
-50
-40
-30

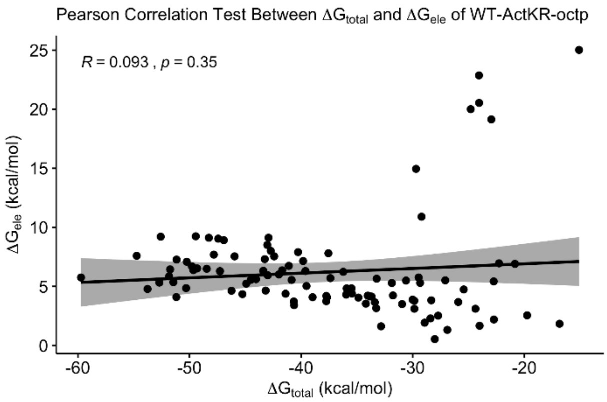
Pearson Correlation Test Between ΔG_{total} and ΔG_{ele} of WT-ActKR-octp
R = 0.093 , p = 0.35
ΔG_{ele} (kcal/mol)
ΔG_{total} (kcal/mol)
25
20
15
10
5
0
-60
-50
-40
-30
-20

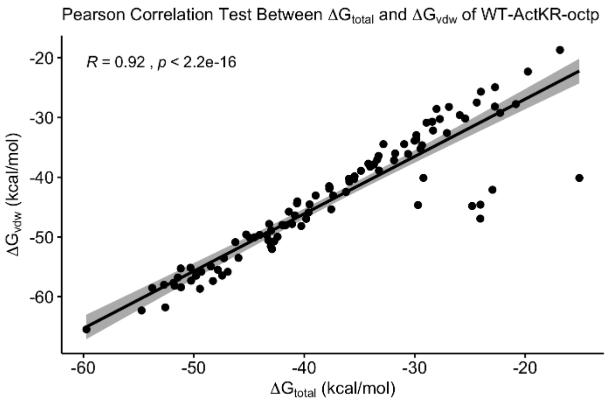
Pearson Correlation Test Between ΔG_{total} and ΔG_{vdw} of WT-ActKR-octp
R = 0.92 , p < 2.2e-16
ΔG_{vdw} (kcal/mol)
ΔG_{total} (kcal/mol)
-20
-30
-40
-50
-60
-60
-50
-40
-30
-20

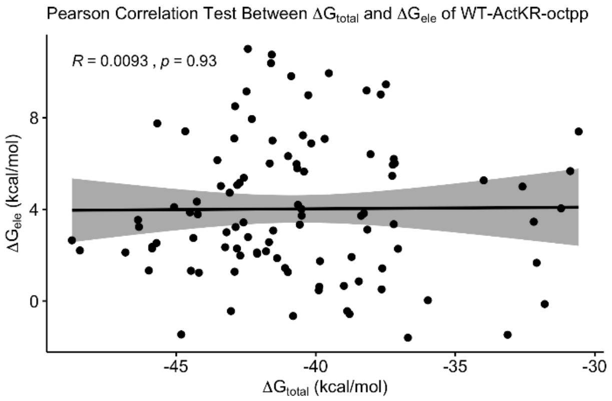
Pearson Correlation Test Between ΔG_{total} and ΔG_{ele} of WT-ActKR-octpp
R = 0.0093 , p = 0.93
ΔG_{ele} (kcal/mol)
ΔG_{total} (kcal/mol)
8
4
0
-45
-40
-35
-30

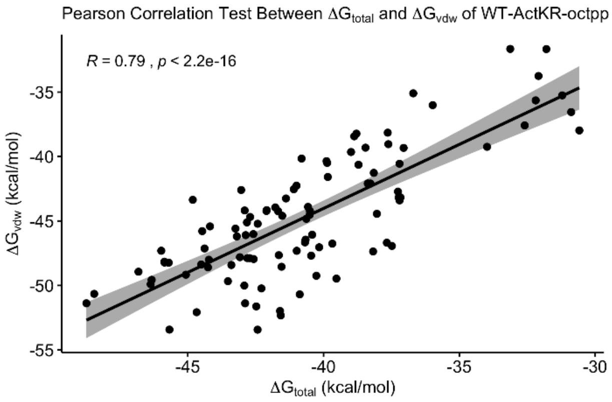
Pearson Correlation Test Between ΔG_{total} and ΔG_{vdw} of WT-ActKR-octpp
R = 0.79 , p < 2.2e-16
ΔG_{vdw} (kcal/mol)
ΔG_{total} (kcal/mol)
-35
-40
-45
-50
-55
-45
-40
-35
-30

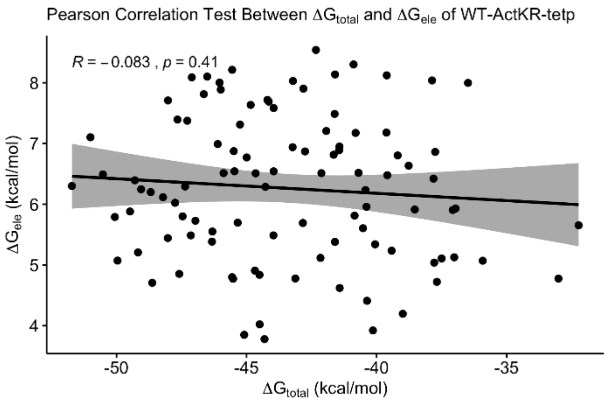
Pearson Correlation Test Between ΔG_total and ΔG_ele of WT-ActKR-tetp
R = −0.083 , p = 0.41
ΔG_ele (kcal/mol)
ΔG_total (kcal/mol)
4
5
6
7
8
-50
-45
-40
-35

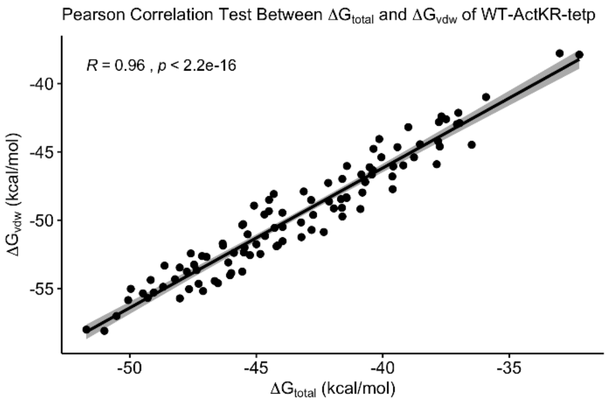
Pearson Correlation Test Between ΔG_total and ΔG_vdw of WT-ActKR-tetp
R = 0.96 , p < 2.2e-16
ΔG_vdw (kcal/mol)
ΔG_total (kcal/mol)
-40
-45
-50
-55
-50
-45
-40
-35

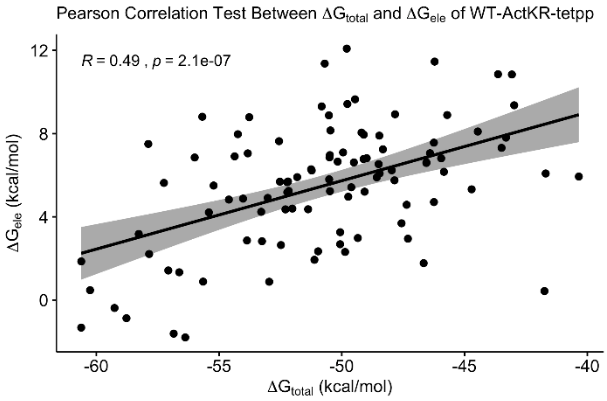
Pearson Correlation Test Between ΔG_total and ΔG_ele of WT-ActKR-tetpp
R = 0.49 , p = 2.1e-07
ΔG_ele (kcal/mol)
ΔG_total (kcal/mol)
12
8
4
0
-60
-55
-50
-45
-40

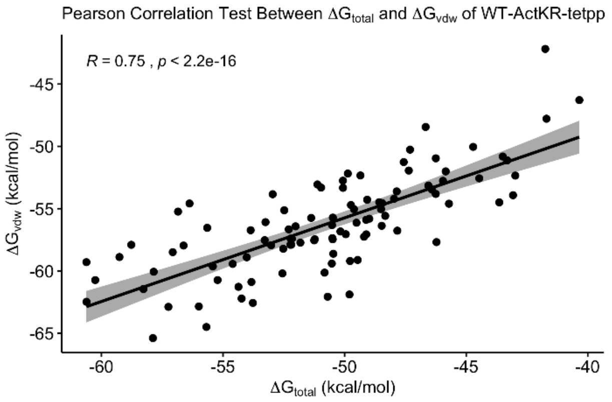
Pearson Correlation Test Between ΔG_total and ΔG_vdw of WT-ActKR-tetpp
R = 0.75 , p < 2.2e-16
ΔG_vdw (kcal/mol)
ΔG_total (kcal/mol)
-45
-50
-55
-60
-65
-60
-55
-50
-45
-40

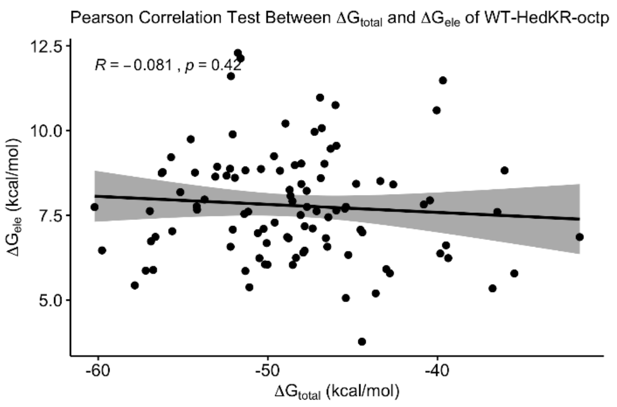
Pearson Correlation Test Between ΔGtotal and ΔGele of WT-HedKR-octp
R = −0.081 , p = 0.42
ΔGele (kcal/mol)
ΔGtotal (kcal/mol)

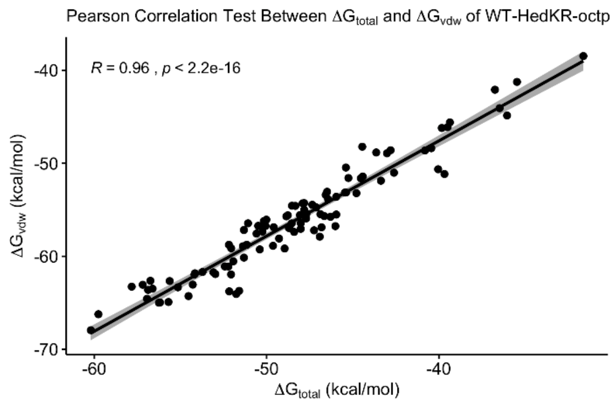
Pearson Correlation Test Between ΔGtotal and ΔGvdw of WT-HedKR-octp
R = 0.96 , p < 2.2e-16
ΔGvdw (kcal/mol)
ΔGtotal (kcal/mol)

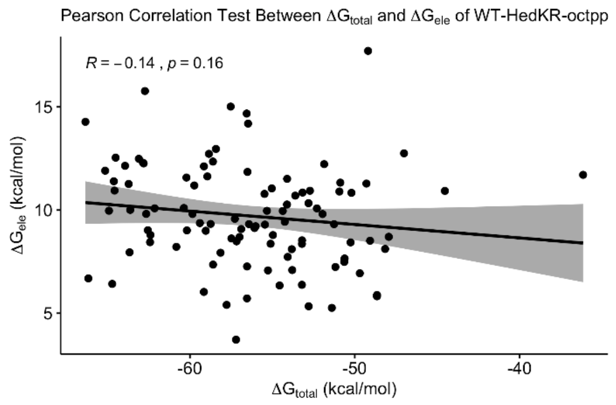
Pearson Correlation Test Between ΔGtotal and ΔGele of WT-HedKR-octpp
R = −0.14 , p = 0.16
ΔGele (kcal/mol)
ΔGtotal (kcal/mol)

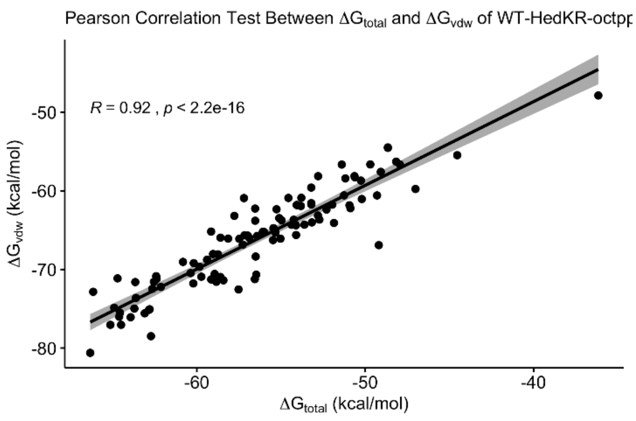
Pearson Correlation Test Between ΔGtotal and ΔGvdw of WT-HedKR-octpp
R = 0.92 , p < 2.2e-16
ΔGvdw (kcal/mol)
ΔGtotal (kcal/mol)

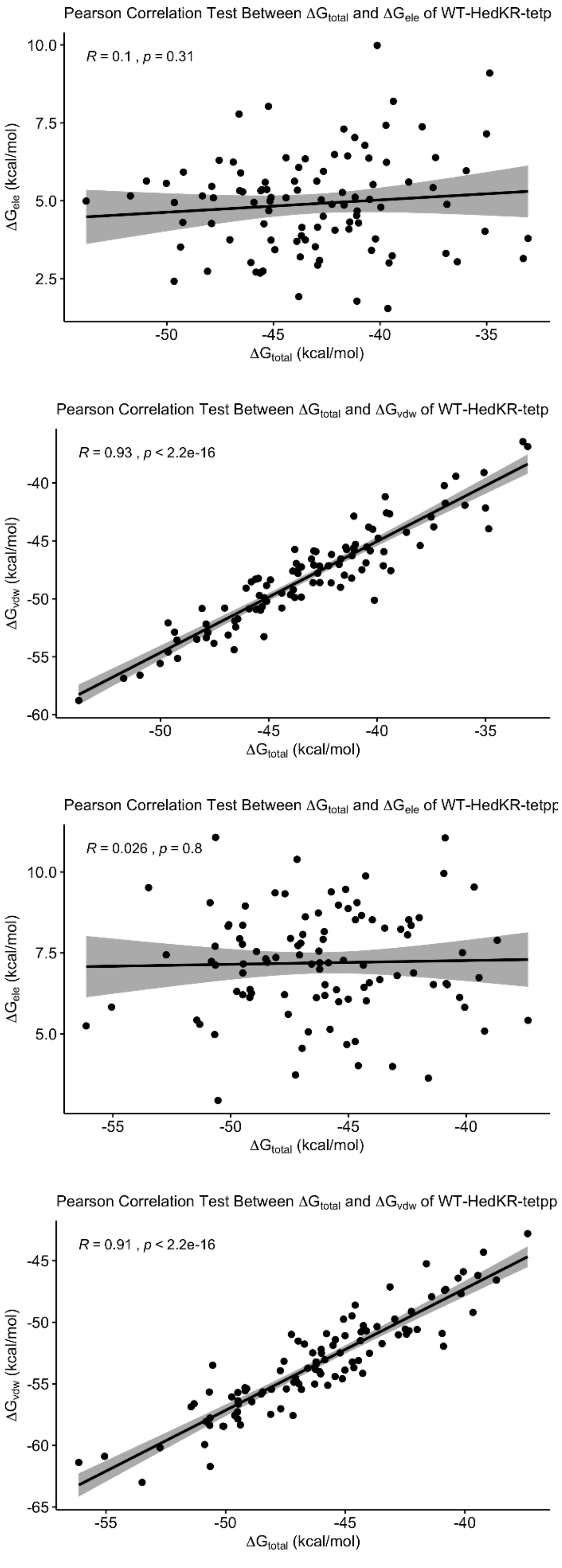

Figure S7. Framewise Pearson correlation test between ΔG_{total} with ΔG_{vdw} or ΔG_{ele} of each KR-ligand pair. ΔG_{total} is the total binding free energy; ΔG_{vdw} is non-electrostatic binding free energy; ΔG_{ele} is electrostatic binding free energy

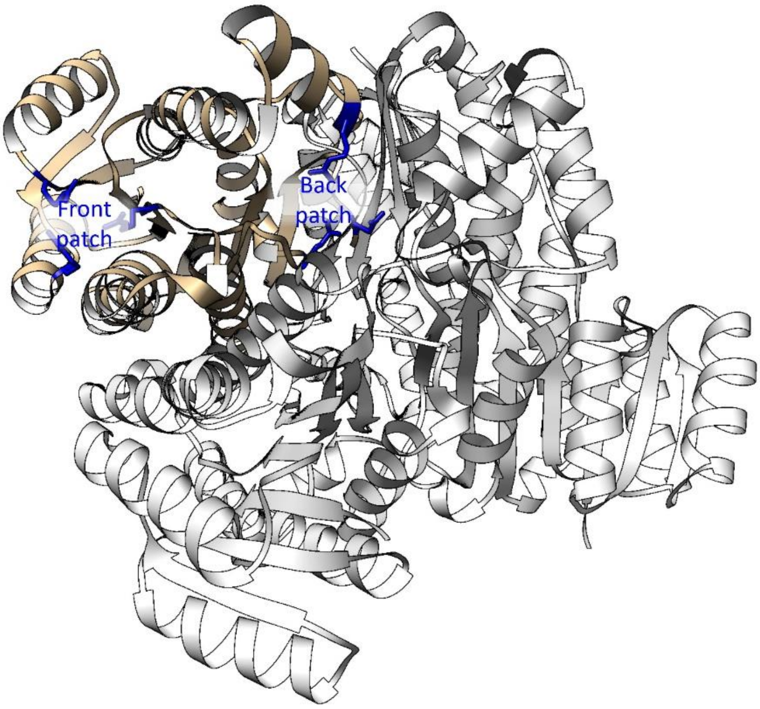

Figure S8. The position of the front patch and back patch in a native *Act*KR tetramer. Only the front patches (left) are exposed to the outer surface, while the back patches (right) are buried inside the interface between monomers, potentially occluding ACP binding. Front and back patches are displayed in blue.
